# Use of Mobile Technologies for Smoking Cessation Among Smokers and Former Smokers: Systematic Review

**DOI:** 10.2196/83072

**Published:** 2026-04-30

**Authors:** Meret Lakeberg, Falk Hoffmann, Jannik Ohmes, Birte Berger-Höger, Jasmin Helbach

**Affiliations:** 1 Department of Health Services Research, School of Medicine and Health Sciences, Carl von Ossietzky Universität Oldenburg, Ammerländer Heerstraße 114-118, Oldenburg, 26129, Germany, +49 441 798 2206; 2 Leibniz Science Campus, Digital Public Health Bremen, Bremen, Germany; 3 Faculty 11 Human and Health Sciences, University of Bremen, Institute for Public Health and Nursing Research, Bremen, Germany

**Keywords:** smoking cessation, mHealth, digital health, quitting smoking, tobacco

## Abstract

**Background:**

In an era of widespread mobile phone usage, digital public health interventions offer a new cost-effective way of improving public health. In the context of smoking cessation, studies indicate that mobile technologies have the potential to support individuals to quit smoking. However, there is no systematic synthesis of how often they are used by smokers and former smokers.

**Objective:**

The aim of this study is to assess the prevalence of mobile technology use for smoking cessation among smokers and former smokers and to examine their intention to use.

**Methods:**

MEDLINE via PubMed, Embase, and PsycInfo were searched from inception to February 13, 2025. Studies were eligible if they reported how often smokers and former smokers in high-income countries used mobile technologies for smoking or vaping cessation. Study quality was assessed using the Joanna Briggs Institute tool for prevalence studies. Data synthesis was conducted narratively.

**Results:**

Twenty-seven cross-sectional studies were included, 25 on smoking and 2 on vaping cessation. The 25 studies on smoking cessation collected data between 2005 and 2024 and comprised 117 to 27,323 participants (mean age 19.9‐50.3 years; n=8). Lifetime prevalences of mobile technology use for smoking cessation ranged between 2.5% and 35.9% (n=8), depending on technology type and population. Period prevalences (0%‐12%; n=4) and point prevalences (1.1%‐10.9%; n=11) were generally lower. Regardless of the prevalence type, the internet was the most frequently used technology (0.8%‐35.9%; n=14). Intention to use mobile technologies for smoking cessation ranged from 19.5% for Twitter to 46.7% for websites (n=2). Of the 2 studies on vaping cessation, 1 presented lifetime prevalence (1.1%‐17.3%), while the other presented period prevalence (5.5%‐6.3%). The intention to use mobile technologies for vaping cessation ranged from 9.7% for web-based programs to 34.6% for apps (n=1). Based on the risk of bias assessment, study quality was heterogeneous, with frequent limitations in sampling procedures, reporting, and reliance on self-reported measures.

**Conclusions:**

This review provides novel insights into the role of mobile technologies in smoking cessation. Evidence indicates that the prevalence of mobile technology use for smoking cessation is low and that disparities in access and engagement exist. However, there is a high intention to use such tools. Therefore, efforts should focus on delivering existing evidence-based tools rather than developing new ones. Included studies were characterized by high methodological variability and poor reporting, so the results must be interpreted with caution. Overall, despite the widespread availability of mobile technologies to support smoking cessation, research on their utilization remains limited.

## Introduction

Tobacco consumption remains the leading preventable cause of disease, disability, and death worldwide, placing a substantial burden on public health systems [1]. Despite decades of prevention efforts, smoking tobacco continues to claim millions of lives each year. The *Global Burden of Disease Study 2019* reported 7.69 million smoking-attributable deaths and over 200 million disability-adjusted life years, with 86.9% of these deaths occurring among current smokers [2]. According to the World Health Organization (WHO) *Tobacco Trends Report 2025*, approximately 1 in 5 adults globally still use tobacco [3]. While smoking prevalence has steadily declined since the 1960s, particularly in high-income countries [4,5], the absolute number of smokers remains high. In Europe, the recent smoking prevalence ranges from 8% in Sweden up to 36% in Greece [6]. Furthermore, the emergence of new tobacco products and technologies, such as electronic cigarettes (e-cigarettes), has led to a new era of nicotine dependency [1]. In 2025, the WHO published its first estimate of the global prevalence of e-cigarettes, indicating that more than 100 million individuals worldwide were currently vaping [3].

Smoking cessation interventions play a key role in the prevention of smoking-related diseases. In 2022, approximately two-thirds of smokers in the United States expressed an intention to quit, and about half of all smokers had made at least 1 quit attempt [7]. Unaided quit attempts resulted in long-term abstinence (6‐12 mo) in approximately 3% to 5% of cases, whereas the use of evidence-based support—such as behavioral counseling combined with nicotine replacement therapy, bupropion, or varenicline—can increase success rates by an additional 7% to 9% [8]. In Germany, data from the DEBRA study (Deutsche Befragung zum Rauchverhalten) show that smoking cessation attempts are common, but only 13% of individuals trying to quit in 2016 to 2019 used at least one evidence-based method [9]. This underlines a persistent gap between available evidence-based interventions and their real-world uptake.

In an era characterized by the widespread use of mobile phones among the global population, with over 82% of individuals above the age of 10 years owning a mobile phone, and a proportion exceeding 95% in high-income countries [10], digital public health interventions offer a new scalable, user-friendly, and cost-effective approach to enhancing public health [11,12], particularly in the domains of prevention and health promotion. In recent years, there has been an increasing integration of digital intervention modalities, encompassing mobile apps, SMS text messaging services, and other web-based interventions, into public health strategies [13-15], including strategies focusing on smoking cessation [16]. The effectiveness of digital smoking cessation interventions has been examined in numerous systematic reviews, which have indicated a promising role for mobile technologies in promoting smoking cessation and abstinence [17-22]. A systematic review examining the cessation of vaping has also demonstrated the efficacy of digital interventions [23]. However, there are no systematic reviews examining the prevalence of the utilization of such aids by smokers and former smokers. Nevertheless, existing evidence shows substantial variability in the prevalence of mobile technology use for smoking cessation across technology types and countries [24-26]. In an analysis of 8 European countries, Papadakis et al [25] reported mobile app use ranging from 0% to 6.2% and use of internet-based resources from 0.75% to 10.2%. Furthermore, user characteristics, including age, sex, and socioeconomic status, appear to be associated with differences in the uptake and engagement with mobile smoking cessation technologies [26-30]. In order to fully exploit the potential of mobile smoking cessation interventions to support behavior change, it is first necessary to systematically synthesize all existing evidence on how often smokers and former smokers use these tools.

Therefore, the primary aim of this systematic review is to provide information on the prevalence of the use of mobile technologies for smoking cessation among smokers and former smokers in high-income countries. A secondary aim is to examine the intention to use these technologies.

## Methods

### Overall study design

This systematic review was conducted in accordance with the Joanna Briggs Institute guideline for systematic reviews of prevalence and incidence [31]. The reporting follows the PRISMA (Preferred Reporting Items for Systematic Reviews and Meta-Analyses) statement [32], and its extension for searches (PRISMA-S; Checklist 1). A study protocol was registered in advance in the PROSPERO (Prospective Register of Systematic Reviews; CRD42025647875).

### Literature Search

MEDLINE (via PubMed), Embase (via Elsevier), and PsycInfo (via EBSCO) were searched from inception to February 13, 2025. No restrictions were applied with regard to either language, study design, or publication date, and no search filters were used. The search strategy was developed by 1 researcher (ML) and reviewed by 2 other researchers (JH and FH). The final search strategies for all databases can be found in Table S1 in Multimedia Appendix 1. To complement the database searches, a forward (citing) and backward (cited) citation analyses of the included studies were performed on May 5, 2025, using Web of Science Core Collection following the TARCiS statement [33]. This analysis was repeated on newly included references until no additional studies could be identified, resulting in a second iteration on May 20, 2025, and a third iteration on May 22, 2025. Registries, online resources, and browsing were not searched, nor were other information sources (eg, contacting authors, experts, and manufacturers) used, as the focus of this review was exclusively on identifying published peer-reviewed studies.

### Eligibility Criteria

We included published observational studies, including cross-sectional studies and cohort studies with data analyzed in a cross-sectional manner. We excluded PhD theses and conference abstracts as well as interventional studies, including randomized controlled trials. Exceptions were made for reports of randomized controlled trials with a cross-sectional analysis of baseline data. No language restrictions were applied.

The study eligibility criteria were defined using the CoCoPop (condition, context, population) approach, which is recommended for reviews of prevalence and incidence data [34].

### Condition

To be included, studies had to report the use of mobile technologies for smoking cessation, addressing smoking of any tobacco product. We included mobile technologies that function as stand-alone tools. The types of mobile technologies assessed in this review included mobile phone apps, SMS text messaging interventions, websites, web-based programs, and social media platforms accessed via mobile devices that provide informational or motivational content related to smoking cessation. Studies that referred to internet use as a smoking cessation resource, without specifying the access device, were included under the category of mobile technologies, acknowledging that a substantial proportion of internet use now occurs via mobile devices [35].

Studies examining the use of interventions that relied exclusively on videoconferencing, telephone calls, or online forums and social media used solely for direct interaction between individuals were excluded.

### Context

For inclusion, the studies had to be conducted in high-income countries according to the OECD (Organisation for Economic Co-operation and Development)–Development Assistance Committee to ensure greater transferability to the German context [36].

### Population

Studies were required to include data on smokers and former smokers as defined by the study authors, with at least 100 individuals in these categories. Studies focusing on specific populations, such as patients with multiple sclerosis or students, were also considered if relevant. To minimize the risk of selection bias, studies exclusively involving individuals who had previously used a specific cessation technology were excluded.

### Study Selection

The search results were imported into EndNote (Version 20, Clarivate), where duplicates were removed. For screening, the results were transferred to Rayyan (Qatar Computing Research Institute). For calibration, all reviewers (ML, JH, and JO) independently screened 100 articles based on title and abstract for inclusion or exclusion. After consensus on these records, ML and one other reviewer (JH or JO) independently screened all remaining titles and abstracts. The full texts of all articles that met the inclusion criteria were also assessed independently by ML and one other reviewer (JH or JO). Any discrepancies were resolved by consensus or, if necessary, by consultation with the third reviewer.

### Data Extraction

The data were extracted directly into the results tables by ML and were subsequently verified by the second reviewer (JO). Discrepancies were resolved through discussion or by the third reviewer (JH). The following items were extracted: study characteristics, characteristics of the study population, definition of smokers, prevalences of mobile technology use for smoking cessation, age- and sex-stratified prevalences of mobile technology use for smoking cessation, types of mobile technology, and prevalences of the intention to use mobile technologies.

### Quality Assessment

The quality of the included studies was independently assessed by two reviewers (ML and JO) using the Joanna Briggs Institute’s critical appraisal checklist for studies reporting prevalence data [37], which was adapted for our research question (Table S2 in Multimedia Appendix 1). The quality assessment was piloted to calibrate the reviewers using 2 studies. Any disagreement was resolved by discussion or by the third reviewer (JH).

### Data Synthesis

The data were summarized descriptively using a narrative synthesis. Due to substantial variability in the clinical and methodological characteristics of the studies, which would result in noncomparable outcomes and populations, it was deemed inappropriate to conduct a meta-analysis by pooling prevalence across studies [37,38]. Instead, this systematic review aimed to describe this variability and present prevalence estimates at the study level, as well as overall prevalence ranges categorized by prevalence type and mobile technologies. Subgroup analyses were conducted on the basis of sex and age, with the prevalence ranges being described in a descriptive manner.

For studies reporting multiple time points, only the most recent data were included to reflect the most current usage patterns. If multiple articles were based on the same study population, only the most recent publication was included. The quality assessment was carried out for each report.

### Deviation From the Protocol

During the peer-review process, it was recommended that studies assessing populations solely using noncombustible products, such as e-cigarettes or vapers, should not be included. In accordance with this recommendation, the primary focus of the review has now been narrowed to populations smoking tobacco products. However, given that the search and initial review also incorporated a systematic identification of studies focusing on the use of mobile technologies in the context of vaping cessation, we decided that these findings should also be presented briefly, but separated from studies on smoking cessation.

## Results

### Study Selection

**Figure 1.**
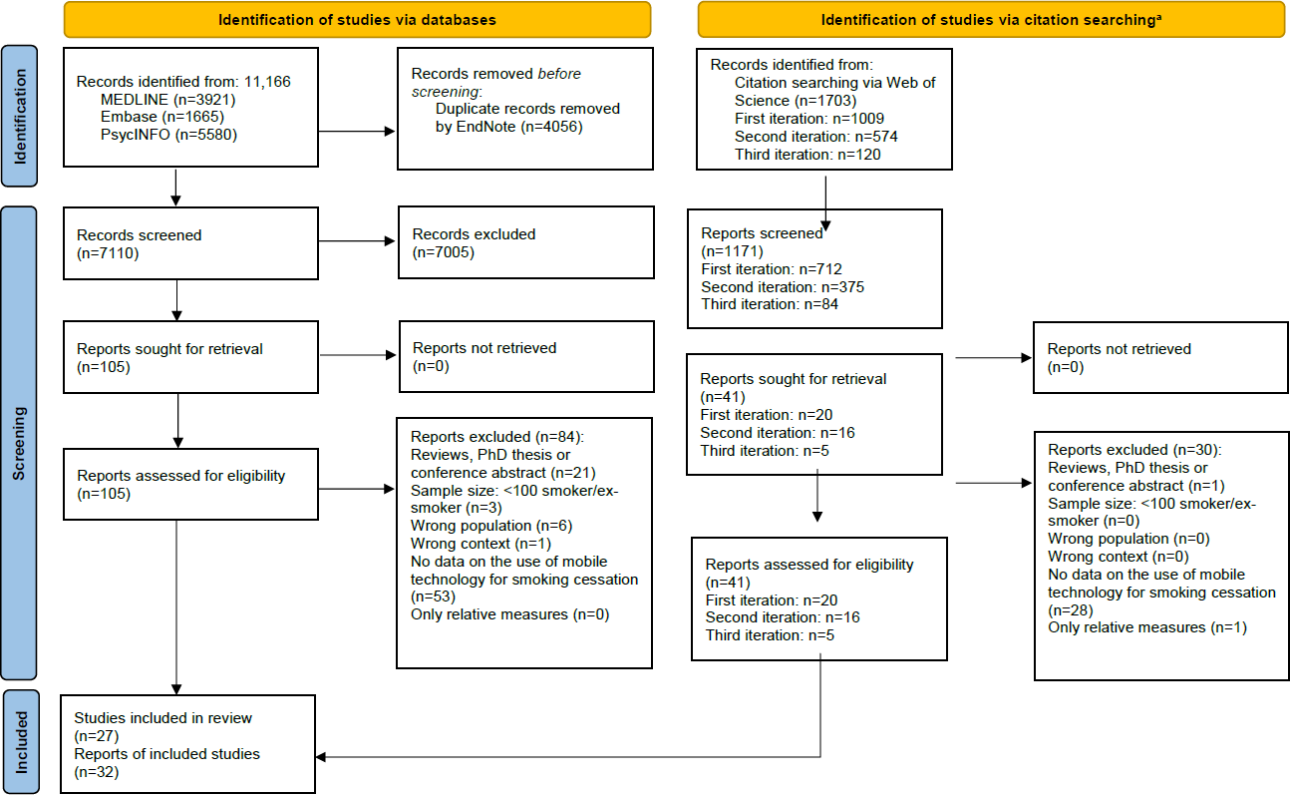
PRISMA (Preferred Reporting Items for Systematic Reviews and Meta-Analyses) study selection diagram. Citation searching was conducted and reported following the TARCIS Statement. The assignment of the reasons for exclusion was done hierarchically in the order presented.

The database search identified 7110 records (Figure 1). After screening the title and abstract, 105 articles were read, and 21 articles reporting on 20 studies were included. A forward-backward citation search was performed on those articles, resulting in the inclusion of 8 additional articles. Based on those 8 articles, a second round of citation analysis was conducted, resulting in 3 additional reports for inclusion. The last round of citation analysis conducted with those 3 reports could not identify any new articles. Overall, 27 studies documented in 32 reports were included in this review. Of these, 25 focused on smoking cessation, while 2 studies focused on vaping cessation. All articles were written in English. The excluded articles are listed in Table S3 in Multimedia Appendix 1 with reasons for exclusion.

### Study and Participant Characteristics of the Studies on Smoking Cessation

The characteristics of the 25 studies focusing on smoking cessation are shown in Table 1. A total of 9 studies were conducted in the United States [39-47], 4 studies were from the United Kingdom [30,48-50], 2 each from Norway [51,52], Canada [53,54], and Australia [55,56], and 1 each from the Netherlands [57] and from Greece [58]. Four studies included several countries in their scope [24-26,59]. All included studies used a cross-sectional design. In terms of data collection methods, 11 studies used online surveys, 4 relied on personal interviews, 4 applied mixed methods, 3 utilized paper-pencil surveys, 2 conducted telephone interviews, and 1 study did not report its data collection method. The types of mobile technologies investigated differed between studies and included websites or general internet use (n=16), smartphone apps (n=8), SMS text messaging programs (n=2), and social media (n=2). Six studies asked for the use of several tools without differentiation, which included web-based programs as well.

The number of participants per study ranged from 117 to 27,323. Although several studies reported the age distribution of their overall study population or subgroups, only 8 out of 25 provided the mean age specifically for smokers, with values ranging from 19.9 to 50.3 years. The proportion of female participants varied between 0% and 77% (n=18). Participants were categorized as either current or former smokers, with the definition of “smoker” varying between studies (Table S4 in Multimedia Appendix 1). Over a third of the included studies (n=10) focused exclusively on smokers who had recently attempted to quit.

**Table 1.** General characteristics of the included studies on smoking cessation (n=25) and vaping cessation (n=2).

Authors and year	Country	Data collection	Year of data	Sample size, n	Population	Mean age (years); female sex (%)
Smoking cessation						
Borrelli et al [24] (2015)	United States/United Kingdom	Online survey	2014	1000	Smokers aged 18 years and older	43.9 years; 55%
Bottorff et al [53] (2016)	Canada	Online survey	2014	117	Smokers, only men^a^ column	39.8 years; 0%
Caraballo et al [39] (2017)	United States	Online survey	2014‐2016	15,943	Smokers aged 18 years and older who made a quit attempt in the last 3 months	N/R^b^; 48.7%
Chevalking et al [57] (2018)	Netherlands	Online survey	2015‐2016	802	Smokers and former smokers^a^	N/R
Curry et al [40] (2007) and companion report: Coups et al [60] (2009)	United States	Personal interview	2005	2747	Smokers aged 18 years and older who made a quit attempt in the past year	N/R
Graham and Amato [61] (2019)	United States	Online survey	2017	1736	Smokers aged 18 years and older	N/R
Gravely et al [59] (2021)	Australia, Canada, England, and United States	Online survey	2020	3614	Smokers, vapers, and former smokers aged 18 years and older who made a quit attempt or quit smoking in the last 2 years	N/R; 53%
Hummel et al [26] (2018)	England, Germany, Greece, Hungary, Netherlands, Poland, Romania, and Spain	Personal interview or online survey	2016	10,683	Smokers aged 18 years and older who made a quit attempt in the past year	N/R
Jackson et al [30] (2019)	United Kingdom	Personal interview	2006‐2018	18,929	Smokers aged 16 years and older who made at least 1 quit attempt in the past year	N/R; 52%
Jackson et al [48] (2022)	United Kingdom	Personal interview	2020‐2021	1550	Smokers and former smokers (smoking during preceding year) aged 18 years and older who made at least 1 quit attempt in the past year	N/R; 50%
Jackson et al [49] (2025)	United Kingdom	Telephone interview	2023‐2024	1642	Smokers and former smokers (smoking during preceding year) aged 16 years and older who made at least 1 quit attempt in the past year	N/R
Jayakumar et al [54] (2019)	Canada	Online survey	2013‐2018	2773	Smokers and former smokers (had quit within last 3 years) aged 18 years and older	N/R; 64%
Kostagiolas et al [58] (2023)	Greece	Paper-pencil survey	2019	150	Smokers aged 18 years and older	N/R; 58%
Li et al [56] (2025)	Australia	Online survey	2017‐2020	1244	Smokers aged 18 years and older from rural/remote locations	43.6 years; 77%
Lund and Lund [52] (2022)	Norway	Telephone interview	2017‐2020	874	Smokers and former smokers aged 20 years and older	50.3 years; 47%
Lund and Lund [51] (2023)	Norway	Online survey	2020	1111	Smokers and former smokers^a^	N/R; 52.8%^c^
Moon et al [42] (2020)	United States	Paper-pencil survey	2018‐2019	227	Smokers with Korean background^a^	N/R; 12.8%
Oliver et al [43] (2018)	United States	Online survey or paper-pencil survey	2013‐2017	180	Smokers aged 18-65 years	N/R
Papadakis et al [25] (2020)	England, Germany, Greece, Hungary, Netherlands, Poland, Romania, and Spain	N/R	2017‐2018	2699	Smokers^a^	N/R
Patterson et al [44] (2021)	United States	Personal interview and telephone interview	2018‐2019	659	Smokers aged 18 years and older	N/R; 43%^d^
Perski et al [50] (2022) and companion reports: Beard et al [62] (2016) and Perski et al [63] (2019)	United Kingdom	Personal interview	2014‐2020	5892	Smokers aged 16 years and older who made a quit attempt in the past year	N/R; 50%
Ramo et al [45] (2015)	United States	Online survey	2010‐2011	570	Smokers aged 18-25 years	19.9 years; 30%
Thrul et al [46] (2021) and companion reports: Mojtabai et al [64](2020) and Soulakova and Crockett [65] (2018)	United States	Personal interview or telephone interview	2010‐2011	27,323	Smokers aged 18 years and older	42.7 years; 46%
Tofighi et al [47](2019)	United States	Paper-pencil survey	2015	157	Smokers enrolled as patients in inpatient detoxification treatment and post discharge (for alcohol and/or heroin)^a^	43.7 years; 9%
Twyman et al [55] (2018)	Australia	Online survey	2012‐2013	646	Smokers and former smokers aged 18 years and older who are financially disadvantaged and made a quit attempt before	39 years; 52%
Vaping cessation						
Dai et al [66] (2023)	United States	Online survey	2021	889	e-Cigarette^e^ users, school grade 6‐12 years, who made quit attempts in the past year^a^	N/R; 51%
Jones et al [67] (2023)	United States	Online survey	N/R	185	Vapers and former vapers aged 14-19 years	16.9 years; 52%

a Included age not displayed.

b N/R: not reported.

c Own calculation based on the numbers provided by the authors in Table 2 (587/1111×100=52.8%).

d Own calculation based on the numbers provided by the authors in Table 2 (280/659×100=42.5%).

e e-Cigarette: electronic cigarette.

### Prevalence of Use of Mobile Technologies for Smoking Cessation

**Figure 2.**
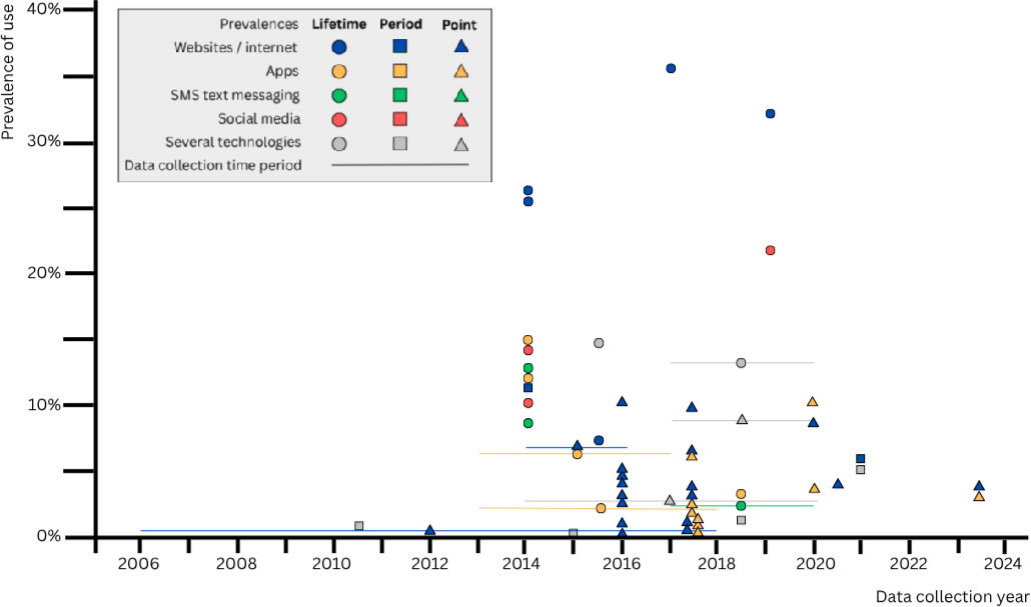
Use of mobile technologies for smoking cessation among smokers and former smokers over time, by prevalence type and technology (n=23; Curry et al [40] and Ramo et al [45] were not included in the figure as no prevalence type was stated or only stratified results were presented).

The data collection periods from the 25 studies on smoking cessation span from 2005 to 2024. A noticeable clustering of studies was observed between 2014 and 2020 (Figure 2), most of which focused on the use of apps and internet-based interventions. After 2021, fewer studies reporting on the use of mobile technologies for smoking cessation were identified.

A total of 8 studies reported the lifetime prevalence of using mobile technology for smoking cessation, which ranged from 2.5% to 35.9% (see Table 2). Four of these studies examined the usage of websites or the internet for smoking cessation, with prevalences ranging from 7.1% up to 35.9%. Mobile app usage ranged from 2.5% to 15.2% (n=4). SMS text messaging programs showed lifetime prevalences between 2.7% and 13.2% (n=2). Two studies presented data on social media use. While 1 study reported a combined prevalence of 22% for social media, another study differentiated between Twitter and Facebook and reported prevalences between 7.6% and 14.8%.

Period prevalence, covering usage within the past several months to the last 2 years, was assessed in 4 studies and ranged from 0% to 12%. Three studies examining the use of websites or the internet for smoking cessation were identified, with 1 study reporting a prevalence of 12%, while the other 2 observed prevalences of less than 2%. One study presented a combined prevalence for apps or SMS text messaging, showing that these tools were never used.

Point prevalence, referring to use during the most recent quit attempt, was reported in 11 studies, with prevalence ranging from 1.1% to 10.9%. For websites and internet use, the prevalences of use ranged from 0% to 10.9% (n=8). Four studies examined smartphone app use, which ranged between 0% and 10.7%.

Two studies reported more than 1 type of prevalence, and 1 study did not specify the type of prevalence assessed.

**Table 2.** Prevalence of the use of different mobile technology tools for smoking cessation among smokers and former smokers, categorized by lifetime, period, and point prevalence.

Authors and year	Country (Sample size, n)	Year of data	Type and prevalence of mobile technology use for smoking cessation, %
Lifetime prevalences			
Borrelli et al [24] (2015)	United States (n=500) United Kingdom (n=500)	2014	United States Websites: 26.2% Text messaging program: 13.2% Apps: 15.2% Twitter: 12.2% Facebook: 14.8% United Kingdom Website: 25.6% Text messaging program: 8.8% Apps: 12.8% Twitter: 7.6% Facebook: 10.4%
Chevalking et al [57] (2018)	Netherlands (n=802)	2015‐2016	Web-based program or apps: 15.5%^a^ Websites: 7.1%^b^
Graham and Amato [61] (2019)	United States (n=1736)	2017	Websites: 35.9%^c^
Jayakumar et al [54] (2020)	Canada (n=2773)	2013‐2018	Apps: 2.5%
Kostagiolas et al [58] (2023)	Greece (n=150)	2019	Internet search engines: 33.3%^d^ Social media (Facebook, Twitter, Blogs): 22.0%^e^ Public institutions websites: 18.7%^f^ Medical websites: 23.3%^g^
Li et al [56] (2025)	Australia (n=1244)	2017‐2020	Online services: 13.5% Text messaging program: 2.7%
Moon et al [42] (2020)	United States (n=227)	2018‐2019	Apps: 3.5%
Oliver et al [43] (2018)	United States (n=180)	2013‐2017	Apps: 6.1%
Period prevalences			
Bottorff et al [53] (2016) (Period prevalence: last 3 months)	Canada (n=117)	2014	Websites: 12%
Patterson et al [44] (2021) (Period prevalence: last 2 years)	United States (n=659)	2018‐2019	Internet/web-based program: 1.7%^h^ Videos: 0.8%^i^
Thrul et al [46] (2021) (Period prevalence: past year)	United States (n=27,323)	2010‐2011	Internet/websites/web-based programs: 0.8%^c^
Tofighi et al [47] (2019) (Period prevalence: past year)	United States (n=157)	2015	Apps or text messaging tool: 0%
Point prevalences (for last quit attempt)			
Caraballo et al [39] (2017)	United States (n=15,943)	2014‐2016	Websites: 7.1%^c^
Curry et al [40] (2007)	United States (n=2747)	2005	Internet: N/R^j^
Gravely et al [59] (2021)	Australia, Canada, England, United States (n=3614)	2020	Apps: 4.6%^c^ Websites: 8.5%^c^
Hummel et al [26] (2018)	England (n=3536) Germany (n=1003) Greece (n=1000) Hungary (n=1000) Netherlands (n=1136) Poland (n=1006) Romania (n=1001) Spain (n=1001)	2016	Internet England: 10.9%^c^ Germany: 4.8%^c^ Greece: 0.4%^c^ Hungary: 2.7%^c^ Netherlands: 5.7%^c^ (period prevalence: last 6 months) Poland: 5.7%^c^ Romania: 3.2%^c^ Spain: 0%^c^
Jackson et al [30] (2019)	United Kingdom (n=18,929)	2006‐2018	Websites: 1.1%
Jackson et al [48] (2022)	United Kingdom (n=1550)	2020‐2021	Websites: 4.1%
Jackson et al [49] (2025)	United Kingdom (n=1642)	2023‐2024	Websites: 4.6%^c^ Apps: 3.6%^c^
Lund and Lund [52] (2022)	Norway (n=874)	2017‐2020	Web/mobile: 9.2%
Lund and Lund [51] (2023)	Norway (n=1111)	2020	Apps: 10.7%
Papadakis et al [25] (2020)	England (n=1482) Germany (n=185) Greece (n=157) Hungary (n=127) Poland (n=179) Romania (n=284) Spain (n=285)	2017‐2018	Apps/Internet: England: 6.2%^c^/10.2^c^ Germany: 4.2%^c^/6.7^c^ Greece: 0.0%^c^/0.7^c^ Hungary: 1.3%^c^/ 2.6^c^ Poland: 0.8%^c^/1.8^c^ Romania: 0.4%^c^/1.7^c^ Spain: 0.9%^c^/0.3^c^
Perski et al [50] (2022)	United Kingdom (n=5892)	2014‐2020	Digital aid (websites, smartphone apps, and other): 2.5%
Prevalence type not reported or more than one prevalence type			
Ramo et al [45] (2015) (Prevalence type not reported)	USA (n=570)	2010‐2011	Internet: 2%
Twyman et al [55] (2018) (Life-time prevalence in current smokers, point prevalence in former smokers)	Australia (n=646)	2012‐2013	Internet: 1%

a Own calculation based on the numbers provided by Chevalking et al [57] on page 4 (124/802 × 100 = 15.5%).

b Own calculation based on the numbers provided by Chevalking et al [57] on page 4 (57/802 × 100 = 7.1%).

c Weighted results.

d Own calculation based on the numbers provided by Kostagiolas et al [58] in Multimedia Appendix 1 [(26 + 24)/150 × 100 = 33.3%].

e Own calculation based on the numbers provided by Kostagiolas et al [58] in Multimedia Appendix 1 [(20 +13)/150 ×100 = 22.0%].

f Own calculation based on the numbers provided by Kostagiolas et al [58] in Multimedia Appendix 1 [(13 + 15)/150 × 100 = 18.7%].

g Own calculation based on the numbers provided by Kostagiolas et al [58] in Multimedia Appendix 1 [(15 + 20)/150 × 100 = 23.3%].

h Own calculation based on the numbers provided by Patterson et al [44] in Table 2 [(3 + 8)/659 × 100 = 1.7%].

i Own calculation based on the numbers provided by Patterson et al [44] in Table 2 [(1 + 4)/659 × 100 = 0.8%].

j N/R: not reported.

### Prevalence of Use of Mobile Technologies for Smoking Cessation, Stratified by Sex and Age

A total of 7 studies reported sex-stratified prevalences of mobile technology use for smoking cessation (Table S5 in Multimedia Appendix 1). Of these, 1 study focused on lifetime prevalences, 2 on period prevalences, and 5 on point prevalences. The study on lifetime prevalence and both studies on period prevalences found comparable usage patterns between males and females. Of the 5 studies that reported point prevalences, 3 did not demonstrate any sex-specific differences in utilization. One study presented higher prevalences of app utilization in females, and 1 study identified significant sex differences in internet utilization that varied across different countries. While males reported higher internet use for smoking cessation in Germany (6.6% males vs 2.9% females), England (14.4% vs 7.3%), Hungary (5.3% vs 0%), and Poland (7.7% vs 3.3%), the opposite was observed in the Netherlands (5.3% vs 6.3%) and Norway (for app use; 5.9% vs 15%), where females reported higher usage.

A total of 5 studies (1 on period prevalence and 4 on point prevalences) reported age-stratified data. Four of the studies assessed the utilization of websites or the internet, and one study assessed the utilization of mobile apps. Regardless of the prevalence type and mobile technology, younger individuals have been found to demonstrate higher utilization of mobile technologies.

### Intention to Use Mobile Technology for Smoking Cessation

Two studies examined the intention to use mobile technologies for smoking cessation (Table 3). One of these studies reported a high intention to use websites (46.7%) and smartphone apps (42.7%). The intention to use SMS text messaging programs was found to be 28.5%, whereas the use of social media, including Twitter and Facebook, was found to be 18.95% and 28.1%, respectively. The other study concentrated on social media in general, reporting an intention to use of 31%.

**Table 3.** The prevalence of smokers’ intentions to use mobile technologies to help them quit smoking in the future.

Authors and year	Country (Sample size, n)	Outcome	Prevalence of intention to use mobile technologies in the future, %
Borrelli et al [24] (2015)	United States and United Kingdom (n=1000)	Future intentions to use technology to quit smoking	Websites: 46.7% SMS text messaging program: 28.5%^a^ Smartphone app: 42.7% Twitter: 19.5%^b^ Facebook: 28.1%^c^
Ramo et al [45] (2015)	United States (n=570)	Intention to use strategies to cut down/quit smoking	Social media: 31%

a Own calculation based on the numbers provided by the authors in Table 5 [(157+128)/1000=28.5%].

b Own calculation based on the numbers provided by the authors in Table 5 [(113+82)/1000=19.5%].

c Own calculation based on the numbers provided by the authors in Table 5 [(152+129)/1000=28.1%].

### Prevalence of Use and Intention to Use Mobile Technologies for Vaping Cessation

A total of 2 studies were identified that focus on vaping cessation [66,67]. The characteristics of these studies are presented in Table 2. Both studies were conducted in the United States, employed a cross-sectional design, and used an online survey for data collection. One study presented lifetime prevalences of the use of SMS text messaging programs (17.3%), apps (12.4%), web-based programs (9.7%), and different social media platforms (1.1% to 12.4%; Table S6 in Multimedia Appendix 1). The other study presented period prevalences of the use of the internet (6.3%) and apps or SMS text messaging programs (5.5%). The intention to use mobile technologies for vaping cessation was presented in 1 study (Table S6 in Multimedia Appendix 1), with the highest intention to use mobile apps (34.6%) being followed by SMS text messaging programs (30.8%) and social media (22.8%).

### Quality Assessment of Studies on Smoking Cessation

Of the 25 studies on smoking cessation, approximately half employed an appropriate sampling frame to address the target population (n=1; Table 4) and used appropriate sampling methods (n=13). A suitable sample size was reported in 80% (20/25) of the studies, while 40% (10/25) provided sufficient detail on study methodology. Forty percent (10/25) of the studies demonstrated unclear attrition, while 56% (14/25) reported sufficient sample coverage. All studies relied on self-reported data. The outcome was measured in a standard and reliable manner in 88% (22/25) of the studies, and 80% (20/25) of the studies applied appropriate statistical analyses. Furthermore, 72% (18/25) of the studies did not report the response rate, and 1 study exhibited an inadequate response rate.

The quality of studies varies according to the prevalence types. While only 13% (1/8) of studies reporting lifetime prevalences employed an appropriate sample frame, 25% (1/4) of studies reporting period prevalences and 81% (9/11) of those on point prevalences did so. Furthermore, appropriate sampling methods were employed in 13% (1/8) of studies reporting lifetime prevalences compared to 50% (2/4) of studies reporting period prevalences and 81% (9/11) of studies reporting point prevalences. The sample size was deemed to be adequate in 63% (5/8) of studies reporting lifetime prevalences, 50% (2/4) of studies reporting period prevalences, and 100% (11/11) of studies reporting point prevalences. With regard to the reporting of study methodology, 9% (1/11) of studies on point prevalences were less adequately reported than 50% (4/8) of studies on lifetime prevalences and 75% (3/4) of studies on period prevalences. For all other categories, there is only minor variability in study quality.

**Table 4.** Overview of the quality of the included studies^a^.

	Was the sample frame appropriate to address the target population?	Were study participants sampled in an appropriate way?	Was the sample size adequate?	Were the study subjects and the setting described in detail?	Was the data analysis conducted with sufficient coverage of the identified sample?	Were valid methods used for the identification of the condition?	Was the condition measured in a standard, reliable way for all participants?	Was there appropriate statistical analysis?	Was the response rate adequate, and if not, was the low response rate managed appropriately?
Smoking cessation									
Lifetime prevalence									
Borrelli et al [24] (2015)	No	No	Yes	Yes	No	No	Yes	Yes	Unclear
Chevalking et al [57] (2018)	No	No	Yes	Yes	Unclear	No	Yes	Yes	Unclear
Graham and Amato [61] (2019)	Yes	Yes	Yes	No	Yes	No	Yes	Yes	Unclear
Jayakumar et al [54] (2019)	No	No	Yes	No	Unclear	No	Yes	Yes	Unclear
Kostagiolas et al [58] (2023)	Unclear	Unclear	No	No	Yes	No	Yes	Yes	Yes
Li et al [56] (2025)	No	No	Yes	Yes	Unclear	No	Yes	Yes	Unclear
Moon et al [42] (2020)	No	No	No	No	Yes	No	Yes	Yes	Unclear
Oliver et al [43] (2018)	No	No	No	Yes	Yes	No	No	No	Unclear
Period prevalence									
Bottorff et al [53] (2016)	No	No	No	Yes	Unclear	No	Yes	Yes	Unclear
Patterson et al [44] (2021)	No	Yes	Yes	No	Unclear	No	Yes	Yes	Unclear
Thrul et al [46] (2021)	Yes	Yes	Yes	Yes	Yes	No	No	Yes	Unclear
Mojtabai et al [64] (2020)	Yes	Yes	Yes	Yes	Yes	No	No	Yes	Unclear
Soulakova and Crockett [65] (2016)	Yes	Yes	Yes	No	Yes	No	No	Yes	Unclear
Tofighi et al [47] (2019)	No	No	No	Yes	Unclear	No	Yes	Yes	Unclear
Point prevalence									
Caraballo et al [39] (2017)	Yes	Yes	Yes	No	Unclear	No	Yes	No	Yes
Curry et al [40] (2007)	Yes	Yes	Yes	No	Yes	No	Yes	No	Yes
Coups et al [60] (2009)	Yes	Yes	No	Yes	Yes	No	Yes	No	Yes
Gravely et al [59] (2021)	No	No	Yes	No	Yes	No	Yes	Yes	Unclear
Hummel et al [26] (2018)	Yes	Yes	Yes	No	Unclear	No	No	Yes	Yes
Jackson et al [30] (2019)	Yes	Yes	Yes	No	Yes	No	Yes	Yes	Unclear
Jackson et al [48] (2022)	Yes	Yes	Yes	No	Yes	No	Yes	Yes	Unclear
Jackson et al [49] (2025)	Yes	Yes	Yes	No	Yes	No	Yes	Yes	Unclear
Lund and Lund [52] (2022)	Yes	Yes	Yes	Yes	Yes	No	Yes	Yes	No
Lund and Lund [51] (2023)	No	No	Yes	No	Unclear	No	Yes	No	Unclear
Papadakis et al [25] (2020)	Yes	Yes	Yes	No	Unclear	No	Yes	Yes	Yes
Perski et al [50] (2022)	Yes	Yes	Yes	No	Yes	No	Yes	Yes	Unclear
Beard et al [62] (2016)	Yes	Yes	Yes	No	Yes	No	Yes	Yes	Unclear
Perski et al [63] (2019)	Yes	Yes	Yes	No	Yes	No	Yes	Yes	Unclear
Prevalence type not reported or more than one prevalence type									
Ramo et al [45] (2015)	No	No	Yes	Yes	Yes	No	Yes	No	Unclear
Twyman et al [55] (2018)	No	No	Yes	Yes	Yes	No	Yes	Yes	Yes
Vaping cessation									
** **Lifetime prevalence									
Dai et al [66] (2023)	Yes	Yes	Yes	No	Yes	No	Yes	Yes	Yes
Period prevalence									
Jones et al [67] (2023)	No	No	No	Yes	Yes	No	Yes	Yes	Unclear

a Further information on the quality assessment criteria can be found in Table S2 in Multimedia Appendix 1.

### Quality Assessment of Studies on Vaping Cessation

Of the 2 studies on vaping cessation, 1 study reporting lifetime prevalences employed an appropriate sampling frame as well as appropriate sampling methods and a suitable sample size, whereas the study reporting period prevalences did not. The study methodology was only described in sufficient detail by the study on period prevalence. Both studies had sufficient sample coverage, measured the outcomes in a standard and reliable manner, and employed appropriate statistical analyses but relied on self-reported data.

## Discussion

### Principal Findings

This systematic review identified 25 studies examining smoking and 2 studies focusing on vaping cessation. Studies on smoking cessation have revealed a wide variability in the reported prevalences of mobile technology use for smoking cessation, ranging from 0% to 35.9% within the data collection period spanning from 2005 to 2024. The clinical and methodological variability observed was influenced by the type of prevalence measured, the specific mobile technology examined, the year of data collection, and the population under study. The lifetime prevalences of mobile technology use for smoking cessation (2.5%‐35.9 %; n=8) were found to be higher than the period prevalences (0%‐12%; n=4) and point prevalences (1.1%‐10.9%; n=11). Regardless of the prevalence type, the internet was the most frequently used technology (0.8%‐35.9%; n=14). In some countries, sex differences were observed, with males usually showing higher prevalences than females. In addition, younger people consistently showed a higher usage of mobile tools. The intention to use was much higher than the actual usage, with prevalences up to 46.7% for websites. In the 2 studies on vaping cessation, the prevalence ranged from 1.1% to 17.2%, and the intention to use mobile technologies ranged from 9.7% for web-based programs to 34.6% for apps.

Our results indicate a peak in research activity between 2014 and 2020, which may be influenced by the digital transformation of health care and the growing number of smoking cessation apps and other digital tools, which are often described as promising [11,18]. Digital tools are increasingly incorporated into public health strategies, including those of the WHO [15,16], and there is a discernible social shift in the popularity of digital platforms. However, since 2021, there has been a noticeable decline in available data, which may be due to delays in publication, shifting research priorities, or the disruptive impact of the COVID-19 pandemic on data collection and health research more broadly.

Although we were able to include more than 27 studies, those are characterized by high methodological variability, encompassing variations in target populations, settings and countries, study aims, sampling frames, outcome definitions, recall periods, and the types of mobile technology assessed. Furthermore, the quality of the majority of the included studies was found to be suboptimal, with particular concerns regarding the adequacy of the sample frame, the sampling strategy employed, and the methods used to assess the utilization of smoking cessation tools. A substantial number of studies have categorized a range of digital tools within broad terms (eg, web/mobile [52], digital aids [50], and online services [56]), without clear differentiation, which has limited the interpretability of specific usage patterns. Furthermore, there is often an absence of reporting on clinical data (eg, comorbidities or tobacco dependence), general smoking patterns (eg, the number of cigarettes smoked per day or number of recent quit attempts), and data on individuals’ technical affinities (eg, mobile phone user patterns or use of other eHealth apps). These factors, however, are crucial in predicting the success of mobile technology in facilitating smoking cessation [68]. Only 2 studies focusing on vaping cessation were identified, which is somewhat surprising given that trends in e-cigarette use have shown significant increases in recent years [68], and digital interventions have been shown to be effective for vaping cessation [23].

Overall, the prevalences of mobile technology use for smoking cessation ranged from 0% to 35.9%, with the majority of studies presenting prevalences from less than 15%. Regardless of the prevalence type, the internet was the most frequently used (up to 35.9%). However, in many studies [25,26,40,44-46,55,58], “internet use” for smoking cessation was poorly defined and could include a variety of digital services that may not be considered stand-alone smoking cessation interventions, such as static informational websites, blogs, or online forums. Furthermore, it is often unclear whether access to web-only interventions occurred via mobile devices or desktop computers. However, it is evident that in the current era, over 90% of internet users utilize smartphones to access the internet [35]. Nevertheless, it is important to recognize that this may not have been the case in previous years, and that a significant proportion of users, estimated at over 50%, still use laptops or desktop computers to access the internet [35]. Surprisingly, the use of apps to support smoking cessation remains relatively low (lifetime prevalences 2.5%‐15.2%), despite the steadily increasing number of available smoking cessation apps [69,70]. By 2017, 177 unique apps relevant to smoking cessation were identified in the iPhone App Store, 139 in Google Play Store, 70 in the BlackBerry App Store, and 55 in the Windows Phone Store [70]. Nevertheless, only 4% of the top applications recommended by leading app stores were found to have any scientific support [71], which makes it challenging for users to identify an appropriate tool [72]. Besides smoking cessation apps, social media platforms represent another promising approach, with a lifetime prevalence of use reaching up to 22.0%. Notwithstanding, only 2 studies [24,58] were identified that examined the prevalence of social media use for smoking cessation, so these numbers must be interpreted with caution. It is evident that social media platforms, which boast over 5 billion users worldwide and high penetration rates in Europe, ranging from 70.4% in Eastern Europe to 81.7% in Northern Europe [73], have the capacity to reach a vast audience. Moreover, reviews have emphasized the feasibility, acceptability, and preliminary effectiveness of social media–based interventions for smoking cessation [74,75]. However, retention rates in such interventions have been reported as low [75], suggesting that social media often functions more as a by-product than as a sustained support tool. A substantial body of evidence supports the notion of digital interventions as a complementary treatment modality for smoking cessation [17-19,70,71,76]. Nevertheless, there remains a paucity of high-quality data regarding the reach of such interventions, and the actual uptake remains low.

The use of mobile technologies for smoking cessation is influenced by a variety of factors, including cultural context, individual age, and socioeconomic status. In 2 of the included studies [25,26], which were based on data from the Europe-wide EUREST-PLUS ITC (European Regulatory Science on Tobacco—International Tobacco Control Project) Study on smoking behavior. Significant differences between countries were identified, and in both studies, the lowest reported use of mobile cessation technologies was found in Greece, where smoking prevalence remains the highest among European countries at 42%. Conversely, the higher prevalence of use was observed in countries with lower smoking prevalence, such as the United Kingdom and the Netherlands (both 12%) [6]. This finding suggests that variations in national smoking norms and tobacco control policies, digital infrastructure, access to health care, and cultural norms may influence both the demand for and the implementation of digital tools. Socioeconomic status and age are also likely to be relevant. Younger persons <40 years of age, compared to older people, showed a consistently higher use of apps, social media, and SMS text messaging programs [26,30,40,46,51]—likely due to their high digital affinity [77]. These patterns may be linked to differences in digital literacy, and especially digital health literacy [78,79], which has been described as a fundamental factor for enabling a successful digital transformation of health care systems and supporting informed decision-making in health contexts [80]. This is particularly relevant for older populations, in which levels of digital competence tend to be lower [81]. Particularly, low use was observed in vulnerable populations, such as financially disadvantaged groups (1%) or people with substance use disorders (0%) [47,55]—possibly reflecting lower motivation to quit or additional barriers to accessing support. Overall, the intention to use mobile technologies for smoking cessation was up to 46.7%, which is somewhat higher than the actual use, indicating a general openness to digital smoking cessation tools. Consequently, efforts should focus less on developing new technologies and more on effectively delivering existing evidence-based tools to populations in need. Addressing disparities in access and engagement is essential to ensure equitable benefits and avoid reinforcing health inequalities. Continuous evaluation of user needs across diverse groups will help guide targeted strategies to enhance uptake and impact.

### Strengths and Limitations

To the best of our knowledge, this is the first systematic review to examine the prevalence of mobile technology use for smoking cessation. While it is possible that some studies were not identified due to the titles and abstracts of these studies not explicitly indicating the inclusion of mobile technologies, the comprehensive search strategy, which was supplemented by repeated forward and backward citation analyses, proved to be highly effective in identifying additional relevant studies.

However, it must be recognized that the present review has certain limitations. First, following the recommendations made during the peer-review process, it was decided to deviate from the established protocol and to narrow the primary focus to populations that smoke tobacco products. Studies assessing populations solely using noncombustible products were now reported only briefly as additional findings. Second, internet use was included as a form of mobile technology. However, a significant number of studies have not provided a detailed definition of internet use, which has resulted in difficulties in classifying it as a stand-alone smoking cessation tool. Moreover, although mobile devices, including smartphones, tablets, and laptops, have become prevalent instruments for accessing the internet in recent years, this has not always been the case. Consequently, the relevance of the internet as a digital smoking cessation aid may have been overestimated. Third, for better comparability, the review focused on high-income countries, which share broadly similar digital infrastructure, access to technology, tobacco control policies, and social norms around smoking; as a result, countries outside this category—such as China, which has a high research output—were not included. Furthermore, we exclusively extracted stratified prevalences on age and sex; yet, other diversity dimensions may also be important. No meta-analysis was conducted due to substantial heterogeneity across studies; yet, the findings provide a broad and current overview of existing prevalence data.

### Conclusion

The prevalence of mobile technology use for smoking cessation is relatively low, and there appears to be disparities in access and engagement, with older and socially disadvantaged groups showing lower prevalences of use. However, the intention to use mobile technologies for smoking cessation is relatively high, indicating a general openness toward such tools. The included studies, however, were characterized by high methodological variability and poor reporting, so the results must be interpreted with caution. It is recommended that future research employ more standardized definitions. Despite the pervasive availability of mobile technologies designed to facilitate smoking cessation and the existence of evidence regarding the efficacy of specific tools, this review indicates that the overall real-world impact of these tools remains limited. Consequently, efforts should concentrate more on evaluating user needs and delivering existing evidence-based tools to populations in need instead of developing new technologies.

## Supplementary material

10.2196/83072 Multimedia Appendix 1 Search strategy, JBI Critical Appraisal Checklist, excluded articles with reason of exclusion, studies definition of smokers, and results stratified by sex/age.

10.2196/83072 Checklist 1 PRISMA-S checklist.

## Abbreviations

CoCoPop: condition, context, population
DEBRA: Deutsche Befragung zum Rauchverhalten
e-cigarette: electronic cigarette
EUREST-PLUS ITC: European Regulatory Science on Tobacco—International Tobacco Control Project
OECD: Organisation for Economic Co-operation and Development
PRISMA: Preferred Reporting Items for Systematic Reviews and Meta-Analyses
WHO: World Health Organization
