# Supplementary material for: Use of Mobile Technologies for Smoking Cessation Among Smokers and Former Smokers: Systematic Review
Source: J Med Internet Res. 2026 Apr 30;28:e83072. doi: 10.2196/83072 (PMC13132022; doi:10.2196/83072)
Supplement: Multimedia Appendix 1 [file jmir-v28-e83072-s001.docx]

**sTable 1 Search strategies**

| **MEDLINE (via PubMed): 3,921 Results** |
| --- |
| **Mobile technologies** |
| ("mobile technolog*"[tiab] OR "mobile device*"[tiab] OR "mobile tool*"[tiab] OR "mobile phone"[tiab] OR "mobile application*"[tiab] OR "cell* phone"[tiab] OR "smartphone*"[tiab] OR "smart phone"[tiab] OR "smart phone app*"[tiab] OR "smartphone app*"[tiab] OR "health app*"[tiab] OR "mobile app*"[tiab] OR "phone-based"[tiab] OR "digital technolog*"[tiab] OR "digital aid*"[tiab] OR "digital health"[tiab] OR "mobile health"[tiab] OR "m-health"[tiab] OR "mhealth"[tiab] OR "digital intervention*"[tiab] OR "digital"[tiab] OR "website*"[tiab] OR "web-based"[tiab] OR "online intervention"[tiab] OR "internet intervention"[tiab] OR "virtual"[tiab] OR "email*"[tiab] OR "e-mail*"[tiab] OR "electronic mail"[tiab] OR "text messag*"[tiab] OR "SMS"[tiab] OR "social media"[tiab] OR "social network*"[tiab] OR "video-based"[tiab] OR "video*"[tiab] OR "smartwatch"[tiab] OR "fitbit"[tiab] OR "wearable"[tiab] OR "Mobile Applications"[mh] OR "Cell Phone"[mh] OR "Fitness Trackers"[mh] OR "Digital Technology"[mh] OR "Internet-Based Intervention"[mh] OR "Text Messaging"[mh] OR "Social Media"[mh] OR "Internet"[mh]) AND |
| **Smoking Cessation** |
| ("smok* cessation"[tiab] OR "quit* smok*"[tiab] OR "stop* smok*"[tiab] OR "ceas* smok*"[tiab] OR "abstain* smok*"[tiab] OR "smok* abstin*"[tiab] OR "smok* reduc*"[tiab] OR "quit* tobacco"[tiab] OR "stop* tobacco"[tiab] OR "tobacco abstin*"[tiab] OR "tobacco cessation"[tiab] OR "tobacco use cessation"[tiab] OR "tobacco reduc*"[tiab] OR "vaping cessation"[tiab] OR "quit* vaping"[tiab] OR "stop* vaping"[tiab] OR "vaping abstin*"[tiab] OR "nicotine cessation"[tiab] OR "nicotine abstin*"[tiab] OR "nicotine reduc*"[tiab] OR "cigar* cessation"[tiab] OR "cigar* abstin*"[tiab] OR "cigar* reduc*"[tiab] OR "stop* cigar*"[tiab] OR "quit* cigar*"[tiab] OR "Smoking Cessation"[mh] OR "Smoking Reduction"[mh] OR "tobacco use cessation"[mh]) |
| Total |
| ("mobile technolog*"[tiab] OR "mobile device*"[tiab] OR "mobile tool*"[tiab] OR "mobile phone"[tiab] OR "mobile application*"[tiab] OR "cell* phone"[tiab] OR "smartphone*"[tiab] OR "smart phone"[tiab] OR "smart phone app*"[tiab] OR "smartphone app*"[tiab] OR "health app*"[tiab] OR "mobile app*"[tiab] OR "phone-based"[tiab] OR "digital technolog*"[tiab] OR "digital aid*"[tiab] OR "digital health"[tiab] OR "mobile health"[tiab] OR "m-health"[tiab] OR "mhealth"[tiab] OR "digital intervention*"[tiab] OR "digital"[tiab] OR "website*"[tiab] OR "web-based"[tiab] OR "online intervention"[tiab] OR "internet intervention"[tiab] OR "virtual"[tiab] OR "email*"[tiab] OR "e-mail*"[tiab] OR "electronic mail"[tiab] OR "text messag*"[tiab] OR "SMS"[tiab] OR "social media"[tiab] OR "social network*"[tiab] OR "video-based"[tiab] OR "video*"[tiab] OR "smartwatch"[tiab] OR "fitbit"[tiab] OR "wearable"[tiab] OR "Mobile Applications"[mh] OR "Cell Phone"[mh] OR "Fitness Trackers"[mh] OR "Digital Technology"[mh] OR "Internet-Based Intervention"[mh] OR "Text Messaging"[mh] OR "Social Media"[mh] OR "Internet"[mh]) AND ("smok* cessation"[tiab] OR "quit* smok*"[tiab] OR "stop* smok*"[tiab] OR "ceas* smok*"[tiab] OR "abstain* smok*"[tiab] OR "smok* abstin*"[tiab] OR "smok* reduc*"[tiab] OR "quit* tobacco"[tiab] OR "stop* tobacco"[tiab] OR "tobacco abstin*"[tiab] OR "tobacco cessation"[tiab] OR "tobacco use cessation"[tiab] OR "tobacco reduc*"[tiab] OR "vaping cessation"[tiab] OR "quit* vaping"[tiab] OR "stop* vaping"[tiab] OR "vaping abstin*"[tiab] OR "nicotine cessation"[tiab] OR "nicotine abstin*"[tiab] OR "nicotine reduc*"[tiab] OR "cigar* cessation"[tiab] OR "cigar* abstin*"[tiab] OR "cigar* reduc*"[tiab] OR "stop* cigar*"[tiab] OR "quit* cigar*"[tiab] OR "Smoking Cessation"[mh] OR "Smoking Reduction"[mh] OR "tobacco use cessation"[mh]) |
| **Embase (via Elsevier)**: **5,580 Results** |
| **Mobile technologies** |
| ('mobile technolog*':ti,ab OR 'mobile device*':ti,ab OR 'mobile tool*':ti,ab OR 'mobile phone':ti,ab OR 'mobile application*':ti,ab OR 'cell* phone':ti,ab OR smartphone*:ti,ab OR 'smart phone':ti,ab OR 'smart phone app*':ti,ab OR 'smartphone app*':ti,ab OR 'health app*':ti,ab OR 'mobile app*':ti,ab OR 'phone based':ti,ab OR 'digital technolog*':ti,ab OR 'digital aid*':ti,ab OR 'digital health':ti,ab OR 'mobile health':ti,ab OR 'm health':ti,ab OR mhealth:ti,ab OR 'digital intervention*':ti,ab OR digital:ti,ab OR 'website*':ti,ab OR 'web based':ti,ab OR 'online intervention':ti,ab OR 'internet intervention':ti,ab OR virtual:ti,ab OR email*:ti,ab OR 'e mail*':ti,ab OR 'electronic mail':ti,ab OR 'text messag*':ti,ab OR sms:ti,ab OR 'social media':ti,ab OR 'social network*':ti,ab OR 'video based':ti,ab OR video*:ti,ab OR smartwatch:ti,ab OR fitbit:ti,ab OR wearable:ti,ab OR 'mobile application'/exp OR 'cell phone'/exp OR 'fitness tracker'/exp OR 'digital technology'/exp OR 'internet-based intervention'/exp OR 'text messaging'/exp OR 'social media'/exp OR 'internet'/exp) AND |
| **Smoking Cessation** |
| ('smok* cessation':ti,ab OR 'quit* smok*':ti,ab OR 'stop* smok*':ti,ab OR 'ceas* smok*':ti,ab OR 'abstain* smok*':ti,ab OR 'smok* abstin*':ti,ab OR 'smok* reduc*':ti,ab OR 'quit* tobacco':ti,ab OR 'stop* tobacco':ti,ab OR 'tobacco abstin*':ti,ab OR 'tobacco cessation':ti,ab OR 'tobacco use cessation':ti,ab OR 'tobacco reduc*':ti,ab OR 'vaping cessation':ti,ab OR 'quit* vaping':ti,ab OR 'stop* vaping':ti,ab OR 'vaping abstin*':ti,ab OR 'nicotine cessation':ti,ab OR 'nicotine abstin*':ti,ab OR 'nicotine reduc*':ti,ab OR 'cigar* cessation':ti,ab OR 'cigar* abstin*':ti,ab OR 'cigar* reduc*':ti,ab OR 'stop* cigar*':ti,ab OR 'quit* cigar*':ti,ab OR 'smoking cessation'/exp OR 'smoking reduction'/exp OR 'tobacco use cessation'/exp) |
| **Total** |
| ('mobile technolog*':ti,ab OR 'mobile device*':ti,ab OR 'mobile tool*':ti,ab OR 'mobile phone':ti,ab OR 'mobile application*':ti,ab OR 'cell* phone':ti,ab OR smartphone*:ti,ab OR 'smart phone':ti,ab OR 'smart phone app*':ti,ab OR 'smartphone app*':ti,ab OR 'health app*':ti,ab OR 'mobile app*':ti,ab OR 'phone based':ti,ab OR 'digital technolog*':ti,ab OR 'digital aid*':ti,ab OR 'digital health':ti,ab OR 'mobile health':ti,ab OR 'm health':ti,ab OR mhealth:ti,ab OR 'digital intervention*':ti,ab OR digital:ti,ab OR 'website*':ti,ab OR 'web based':ti,ab OR 'online intervention':ti,ab OR 'internet intervention':ti,ab OR virtual:ti,ab OR email*:ti,ab OR 'e mail*':ti,ab OR 'electronic mail':ti,ab OR 'text messag*':ti,ab OR sms:ti,ab OR 'social media':ti,ab OR 'social network*':ti,ab OR 'video based':ti,ab OR video*:ti,ab OR smartwatch:ti,ab OR fitbit:ti,ab OR wearable:ti,ab OR 'mobile application'/exp OR 'cell phone'/exp OR 'fitness tracker'/exp OR 'digital technology'/exp OR 'internet-based intervention'/exp OR 'text messaging'/exp OR 'social media'/exp OR 'internet'/exp) AND ('smok* cessation':ti,ab OR 'quit* smok*':ti,ab OR 'stop* smok*':ti,ab OR 'ceas* smok*':ti,ab OR 'abstain* smok*':ti,ab OR 'smok* abstin*':ti,ab OR 'smok* reduc*':ti,ab OR 'quit* tobacco':ti,ab OR 'stop* tobacco':ti,ab OR 'tobacco abstin*':ti,ab OR 'tobacco cessation':ti,ab OR 'tobacco use cessation':ti,ab OR 'tobacco reduc*':ti,ab OR 'vaping cessation':ti,ab OR 'quit* vaping':ti,ab OR 'stop* vaping':ti,ab OR 'vaping abstin*':ti,ab OR 'nicotine cessation':ti,ab OR 'nicotine abstin*':ti,ab OR 'nicotine reduc*':ti,ab OR 'cigar* cessation':ti,ab OR 'cigar* abstin*':ti,ab OR 'cigar* reduc*':ti,ab OR 'stop* cigar*':ti,ab OR 'quit* cigar*':ti,ab OR 'smoking cessation'/exp OR 'smoking reduction'/exp OR 'tobacco use cessation'/exp) |
| **PsycINFO (via EBSCO): 1,665 Results** |
| **Mobile technologies** |
| ((TI "mobile technolog*" OR AB "mobile technolog*" OR TI "mobile device*" OR AB "mobile device*" OR TI "mobile tool*" OR AB "mobile tool*" OR TI "mobile phone" OR AB "mobile phone" OR TI "mobile application*" OR AB "mobile application*" OR TI "cell* phone" OR AB "cell* phone" OR TI smartphone* OR AB smartphone* OR TI "smart phone" OR AB "smart phone" OR TI "smart phone app*" OR AB "smart phone app*" OR TI "smartphone app*" OR AB "smartphone app*" OR TI "health app*" OR AB "health app*" OR TI "mobile app*" OR AB "mobile app*" OR TI phone-based OR AB phone-based OR TI "digital technolog*" OR AB "digital technolog*" OR TI "digital aid*" OR AB "digital aid*" OR TI "digital health" OR AB "digital health" OR TI "mobile health" OR AB "mobile health" OR TI m-health OR AB m-health OR TI mhealth OR AB mhealth OR TI "digital intervention*" OR AB "digital intervention*" OR TI digital OR AB digital OR TI "website*" OR AB "website*" OR TI web-based OR AB web-based OR TI "online intervention" OR AB "online intervention" OR TI "internet intervention" OR AB "internet intervention" OR TI virtual OR AB virtual OR TI email* OR AB email* OR TI e-mail* OR AB e-mail* OR TI "electronic mail" OR AB "electronic mail" OR TI "text messag*" OR AB "text messag*" OR TI SMS OR AB SMS OR TI "social media" OR AB "social media" OR TI "social network*" OR AB "social network*" OR TI video-based OR AB video-based OR TI video* OR AB video* OR TI smartwatch OR AB smartwatch OR TI fitbit OR AB fitbit OR TI wearable OR AB wearable OR MA "Mobile Applications" OR MA "Cell Phone" OR MA "Fitness Trackers" OR MA "Digital Technology" OR MA "Internet-Based Intervention" OR MA "Text Messaging" OR MA "Social Media" OR MA "Internet")) AND |
| **Smoking Cessation** |
| ((TI "smok* cessation" OR AB "smok* cessation" OR TI "quit* smok*" OR AB "quit* smok*" OR TI "stop* smok*" OR AB "stop* smok*" OR TI "ceas* smok*" OR AB "ceas* smok*" OR TI "abstain* smok*" OR AB "abstain* smok*" OR TI "smok* abstin*" OR AB "smok* abstin*" OR TI "smok* reduc*" OR AB "smok* reduc*" OR TI "quit* tobacco" OR AB "quit* tobacco" OR TI "stop* tobacco" OR AB "stop* tobacco" OR TI "tobacco abstin*" OR AB "tobacco abstin*" OR TI "tobacco cessation" OR AB "tobacco cessation" OR TI "tobacco use cessation" OR AB "tobacco use cessation" OR TI "tobacco reduc*" OR AB "tobacco reduc*" OR TI "vaping cessation" OR AB "vaping cessation" OR TI "quit* vaping" OR AB "quit* vaping" OR TI "stop* vaping" OR AB "stop* vaping" OR TI "vaping abstin*" OR AB "vaping abstin*" OR TI "nicotine cessation" OR AB "nicotine cessation" OR TI "nicotine abstin*" OR AB "nicotine abstin*" OR TI "nicotine reduc*" OR AB "nicotine reduc*" OR TI "cigar* cessation" OR AB "cigar* cessation" OR TI "cigar* abstin*" OR AB "cigar* abstin*" OR TI "cigar* reduc*" OR AB "cigar* reduc*" OR TI "stop* cigar*" OR AB "stop* cigar*" OR TI "quit* cigar*" OR AB "quit* cigar*" OR MA "Smoking Cessation" OR MA "Smoking Reduction" OR MA "Tobacco Use Cessation")) |
| **Total** |
| ((TI "mobile technolog*" OR AB "mobile technolog*" OR TI "mobile device*" OR AB "mobile device*" OR TI "mobile tool*" OR AB "mobile tool*" OR TI "mobile phone" OR AB "mobile phone" OR TI "mobile application*" OR AB "mobile application*" OR TI "cell* phone" OR AB "cell* phone" OR TI smartphone* OR AB smartphone* OR TI "smart phone" OR AB "smart phone" OR TI "smart phone app*" OR AB "smart phone app*" OR TI "smartphone app*" OR AB "smartphone app*" OR TI "health app*" OR AB "health app*" OR TI "mobile app*" OR AB "mobile app*" OR TI phone-based OR AB phone-based OR TI "digital technolog*" OR AB "digital technolog*" OR TI "digital aid*" OR AB "digital aid*" OR TI "digital health" OR AB "digital health" OR TI "mobile health" OR AB "mobile health" OR TI m-health OR AB m-health OR TI mhealth OR AB mhealth OR TI "digital intervention*" OR AB "digital intervention*" OR TI digital OR AB digital OR TI "website*" OR AB "website*" OR TI web-based OR AB web-based OR TI "online intervention" OR AB "online intervention" OR TI "internet intervention" OR AB "internet intervention" OR TI virtual OR AB virtual OR TI email* OR AB email* OR TI e-mail* OR AB e-mail* OR TI "electronic mail" OR AB "electronic mail" OR TI "text messag*" OR AB "text messag*" OR TI SMS OR AB SMS OR TI "social media" OR AB "social media" OR TI "social network*" OR AB "social network*" OR TI video-based OR AB video-based OR TI video* OR AB video* OR TI smartwatch OR AB smartwatch OR TI fitbit OR AB fitbit OR TI wearable OR AB wearable OR MA "Mobile Applications" OR MA "Cell Phone" OR MA "Fitness Trackers" OR MA "Digital Technology" OR MA "Internet-Based Intervention" OR MA "Text Messaging" OR MA "Social Media" OR MA "Internet")) AND ((TI "smok* cessation" OR AB "smok* cessation" OR TI "quit* smok*" OR AB "quit* smok*" OR TI "stop* smok*" OR AB "stop* smok*" OR TI "ceas* smok*" OR AB "ceas* smok*" OR TI "abstain* smok*" OR AB "abstain* smok*" OR TI "smok* abstin*" OR AB "smok* abstin*" OR TI "smok* reduc*" OR AB "smok* reduc*" OR TI "quit* tobacco" OR AB "quit* tobacco" OR TI "stop* tobacco" OR AB "stop* tobacco" OR TI "tobacco abstin*" OR AB "tobacco abstin*" OR TI "tobacco cessation" OR AB "tobacco cessation" OR TI "tobacco use cessation" OR AB "tobacco use cessation" OR TI "tobacco reduc*" OR AB "tobacco reduc*" OR TI "vaping cessation" OR AB "vaping cessation" OR TI "quit* vaping" OR AB "quit* vaping" OR TI "stop* vaping" OR AB "stop* vaping" OR TI "vaping abstin*" OR AB "vaping abstin*" OR TI "nicotine cessation" OR AB "nicotine cessation" OR TI "nicotine abstin*" OR AB "nicotine abstin*" OR TI "nicotine reduc*" OR AB "nicotine reduc*" OR TI "cigar* cessation" OR AB "cigar* cessation" OR TI "cigar* abstin*" OR AB "cigar* abstin*" OR TI "cigar* reduc*" OR AB "cigar* reduc*" OR TI "stop* cigar*" OR AB "stop* cigar*" OR TI "quit* cigar*" OR AB "quit* cigar*" OR MA "Smoking Cessation" OR MA "Smoking Reduction" OR MA "Tobacco Use Cessation")) |

**sTable 2: Quality assessment criteria - JBI Critical Appraisal Checklist for reporting prevalence data**

| 1. Was the sample frame appropriate to address the target population? | |
| --- | --- |
| Yes | Study focusing on the total population |
| No | Study including smokers e.g. only from one clinic or recruited by a market research panel |
| Unclear | Sample frame not described clearly |
| N/A | - |
| 2. Were study participants sampled in an appropriate way? | |
| Yes | All participants considered or systematic sampled |
| No | Convenience sample |
| Unclear | Unclear whether all willing participants were considered or how the sampling was conducted |
| N/A | Administrative data |
| 3. Was the sample size adequate? | |
| Yes | 384+ participants (based on sample size calculation: (1.96²x0.5(1-0.5))/0.05²) |
| No | <384 participants |
| Unclear | Number of included participants not reported and no indication that 384+ participants were included |
| N/A | - |
| 4. Were the study subjects and the setting described in detail? | |
| Yes | Number of included (former) smokers, mean/median age, proportion of females and country reported |
| No | Number of included (former) smokers, mean/median age, proportion of females and country (partially) not reported |
| Unclear | - |
| N/A | - |
| 5. Was the data analysis conducted with sufficient coverage of the identified sample? | |
| Yes | Low attrition, no systematic exclusions or systematic exclusions with clear justification |
| No | Exclusion of relevant subgroups without justification, exclusion with insufficient justification or high attrition not addressed |
| Unclear | No information on exclusions |
| N/A | - |
| 6. Were valid methods used for the identification of the condition? | |
| Yes | Objective data sources as digital usage data |
| No | Self-reported data |
| Unclear | Method of measurement not described |
| N/A | - |
| 7. Was the condition measured in a standard, reliable way for all participants? | |
| Yes | Same measurement tool or question used for all participants, administered by e.g. trained interviewers or through automated systems |
| No | Inconsistent methods within the study, or measurement varies across groups without justification |
| Unclear | No information on how data were collected or by whom |
| N/A | - |
| 8. Was there appropriate statistical analysis? | |
| Yes | Prevalence clearly calculated (point, period, or lifetime) with numerator and denominator, weighted results with confidence intervals |
| No | Numerator or denominator not reported |
| Unclear | Analysis not described adequately |
| N/A | - |
| 9. Was the response rate adequate, and if not, was the low response rate managed appropriately? | |
| Yes | Response ≥ 60%, or < 60% but addressed appropriately (e.g., with weighting or imputation) |
| No | Response < 60% with no evidence of management |
| Unclear | Response rate not reported |
| N/A | Administrative data |
| Abbreviation: N/A, not applicable  Reference: Munn, Z., Moola, S., Lisy, K., Riitano, D., & Tufanaru, C. (2015). Methodological guidance for systematic reviews of observational epidemiological studies reporting prevalence and cumulative incidence data. *International journal of evidence-based healthcare*, *13*(3), 147–153. | |

**sTable 3 – Excluded articles with reason for exclusion**

| **Articles** | **Reason for exclusion** |
| --- | --- |
| Adams, K., Paasse, G., & Clinch, D. (2011). Peer-support preferences and readiness-to-change behaviour for chronic disease prevention in an urban indigenous population. *Australian Social Work*, *64*(1), 55-67. https://doi.org/doi:10.1080/0312407X.2010.541269 | Sample size (<100 smokers) |
| Ajay, V. S., Praveen, P. A., Millett, C., Kinra, S., & Prabhakaran, D. (2012). Role of mobile phone technology in tobacco cessation interventions. *Global Heart*, *7*(2), 167-174. https://doi.org/doi:10.1016/j.gheart.2012.03.004 | Review, editorials, PhD theses, conference abstracts |
| Akiyama, O., Nakamura, M., & Tabuchi, T. (2018). Awareness of harm to others from secondhand smoke and smokers' interest in smoking cessation. *[Nihon koshu eisei zasshi] Japanese journal of public health*, *65*(11), 655-665. https://doi.org/doi:10.11236/jph.65.11_655 | No data on the use of mobile technology for smoking cessation |
| Al Shahrani, A. S., Almudaiheem, N. R., Bakhsh, E. M., Sarhan, N. T., Aldossari, F. S., & Bin Huzeim, B. A. (2021). Understanding Smoking Behavior among Physicians in Riyadh, Saudi Arabia: A Cross-Sectional Study. *Tob Use Insights*, *14*, 1179173x211006652. https://doi.org/doi:10.1177/1179173x211006652 | No data on the use of mobile technology for smoking cessation |
| Al-Hamdani, M., Davidson, M., Bird, D., Hopkins, D. B., & Smith, S. (2023). Learning from their experiences: Strategies used by youth and young adult ex-vapers. *Journal of Substance Use and Addiction Treatment*, *149*. https://doi.org/doi:10.1016/j.josat.2023.209038 | No data on the use of mobile technology for smoking cessation |
| Al-Khadher, M. A. (2023). Assessment of the use and awareness of health applications among the public in Najran City, Saudi Arabia: A cross sectional study. *Rawal Medical Journal*, *48*(4), 1039-1044. https://doi.org/doi:10.5455/RMJ.20230914102028 | Wrong population (e.g. pre-selected by app use) |
| Al-Tabakha, M. M., Alomar, M. J., Awad, M. N. G., & Fahelelbom, K. M. S. (2019). Smoking Patterns and Willingness to Quit: A Cross-sectional Study in Al Ain City, UAE. *J Pharm Bioallied Sci*, *11*(3), 276-283. https://doi.org/doi:10.4103/jpbs.JPBS_30_19 | No data on the use of mobile technology for smoking cessation |
| Alqahtani, A. S. (2022). Awareness of current mobile apps for smoking cessation among the dental and medical practitioners in Saudi Arabia. *European Review for Medical and Pharmacological Sciences*, *26*(18), 6561-6568. https://doi.org/doi:10.26355/eurrev_202209_29754 | No data on the use of mobile technology for smoking cessation |
| Aminorroaya, A., Dhingra, L. S., Nargesi, A. A., Oikonomou, E. K., Krumholz, H. M., & Khera, R. (2023). Use of Smart Devices to Track Cardiovascular Health Goals in the United States. *JACC Adv*, *2*(7). https://doi.org/doi:10.1016/j.jacadv.2023.100544 | No data on the use of mobile technology for smoking cessation |
| Aminorroaya, A., Dhingra, L. S., Oikonomou, E., Nargesi, A. A., & Khera, R. (2022). A Nationally Representative Evaluation of Patients With Established Cardiovascular Disease and Cardiovascular Risk Factors Achieving Health Goals With Electronic Devices in the United States. *Circulation*, *146*. https://doi.org/doi:10.1161/circ.146.suppl_1.15516 | Review, editorials, PhD theses, conference abstracts |
| Andrews, L., Cacho-Elizondo, S., Drennan, J., & Tossan, V. (2013). Consumer Acceptance of an SMS-Assisted Smoking Cessation Intervention: A Multicountry Study. *Health Marketing Quarterly*, *30*(1), 47-62. https://doi.org/doi:10.1080/07359683.2013.758015 | No data on the use of mobile technology for smoking cessation |
| Bachmann, M. S., Znoj, H., & Brodbeck, J. (2012). Smoking behaviour, former quit attempts and intention to quit in urban adolescents and young adults: A five-year longitudinal study. *PUBLIC HEALTH*, *126*(12), 1044-1050. https://doi.org/doi:10.1016/j.puhe.2012.08.006 | No data on the use of mobile technology for smoking cessation |
| Barrera, A. Z., Pérez-Stable, E. J., Delucchi, K. L., & Muñoz, R. F. (2009). Global reach of an Internet smoking cessation intervention among Spanish- and English-speaking smokers from 157 countries. *Int J Environ Res Public Health*, *6*(3), 927-940. https://doi.org/doi:10.3390/ijerph6030927 | No data on the use of mobile technology for smoking cessation |
| Berg, C. J., Romm, K. F., Patterson, B., Wysota, C., & Abroms, L. C. (2021). Appeal of novel cessation intervention approaches among young-adult users of traditional and alternative tobacco products. *Tob Use Insights*, *14*, 1179173x211041123. https://doi.org/doi:10.1177/1179173x211041123 | No data on the use of mobile technology for smoking cessation |
| Bold, K. W., Deng, Y., Dziura, J., Porter, E., Sigel, K. M., Yager, J. E., Ledgerwood, D. M., Bernstein, S. L., & Edelman, E. J. (2022). Practices, attitudes, and confidence related to tobacco treatment interventions in HIV clinics: a multisite cross-sectional survey. *Transl Behav Med*, *12*(6), 726-733. https://doi.org/doi:10.1093/tbm/ibac022 | Wrong population (e.g. pre-selected by app use) |
| Borland, R., Li, L., Driezen, P., Wilson, N., Hammond, D., Thompson, M. E., Fong, G. T., Mons, U., Willemsen, M. C., McNeill, A., Thrasher, J. F., & Cummings, K. M. (2012). Cessation assistance reported by smokers in 15 countries participating in the International Tobacco Control (ITC) policy evaluation surveys. *Addiction*, *107*(1), 197-205. https://doi.org/doi:10.1111/j.1360-0443.2011.03636.x | No data on the use of mobile technology for smoking cessation |
| Borrelli, B., Busch, A. M., & Trotter, D. R. M. (2013). Methods Used to Quit Smoking by People With Physical Disabilities. *REHABILITATION PSYCHOLOGY*, *58*(2), 117-123. https://doi.org/doi:10.1037/a0031577 | No data on the use of mobile technology for smoking cessation |
| Brown, J., Michie, S., Raupach, T., & West, R. (2013). Prevalence and characteristics of smokers interested in internet-based smoking cessation interventions: cross-sectional findings from a national household survey. *J Med Internet Res*, *15*(3), e50. https://doi.org/doi:10.2196/jmir.2342 | No data on the use of mobile technology for smoking cessation |
| Brown-Johnson, C. G., Boeckman, L. M., White, A. H., Burbank, A. D., Paulson, S., & Beebe, L. A. (2018). Trust in Health Information Sources: Survey Analysis of Variation by Sociodemographic and Tobacco Use Status in Oklahoma. *JMIR Public Health Surveill*, *4*(1), e8. https://doi.org/doi:10.2196/publichealth.6260 | No data on the use of mobile technology for smoking cessation |
| Centers for Disease, C., & Prevention. (2006). Use of cessation methods among smokers aged 16-24 years--United States, 2003. *MMWR. Morbidity and mortality weekly report*, *55*(50), 1351-1354. https://doi.org/doi: | Review, editorials, PhD theses, conference abstracts |
| Centers for Disease, C., & Prevention. (2011). Quitting smoking among adults--United States, 2001-2010. *MMWR. Morbidity and mortality weekly report*, *60*(44), 1513-1519. https://doi.org/doi: | No data on the use of mobile technology for smoking cessation |
| Chen, P. C., Chang, L. C., Hsu, C., & Lee, Y. C. (2019). Electronic Cigarette Use and Attempts to Quit Smoking Cigarettes Among Adolescents in Taiwan. *Journal of Adolescent Health*, *64*(1), 99-106. https://doi.org/doi:10.1016/j.jadohealth.2018.07.008 | No data on the use of mobile technology for smoking cessation |
| Cobb, C. O., & Graham, A. L. (2014). Use of non-assigned interventions in a randomized trial of internet and telephone treatment for smoking cessation. *Nicotine Tob Res*, *16*(10), 1289-1297. https://doi.org/doi:10.1093/ntr/ntu066 | No data on the use of mobile technology for smoking cessation |
| Cokkinides, V. E., Halpern, M. T., Barbeau, E. M., Ward, E., & Thun, M. J. (2008). Racial and ethnic disparities in smoking-cessation interventions - Analysis of the 2005 National Health Interview Survey. *AMERICAN JOURNAL OF PREVENTIVE MEDICINE*, *34*(5), 404-412. https://doi.org/doi:10.1016/j.amepre.2008.02.003 | No data on the use of mobile technology for smoking cessation |
| Cunningham, J. A. (2008). Access and interest: two important issues in considering the feasibility of web-assisted tobacco interventions. *J Med Internet Res*, *10*(5), e37. https://doi.org/doi:10.2196/jmir.1000 | No data on the use of mobile technology for smoking cessation |
| Dahne, J., Wahlquist, A. E., Garrett-Mayer, E., Heckman, B. W., Cummings, K. M., & Carpenter, M. J. (2018). State Tobacco Policies as Predictors of Evidence-Based Cessation Method Usage: Results From a Large, Nationally Representative Dataset. *NICOTINE & TOBACCO RESEARCH*, *20*(11), 1336-1343. https://doi.org/doi:10.1093/ntr/ntx192 | No data on the use of mobile technology for smoking cessation |
| de Moor, J. S., Puleo, E., Ford, J. S., Greenberg, M., Hodgson, D. C., Tyc, V. L., Ostroff, J., Diller, L. R., Levy, A. G., Sprunck-Harrild, K., & Emmons, K. M. (2011). Disseminating a smoking cessation intervention to childhood and young adult cancer survivors: baseline characteristics and study design of the partnership for health-2 study. *BMC Cancer*, *11*, 165. https://doi.org/doi:10.1186/1471-2407-11-165 | No data on the use of mobile technology for smoking cessation |
| Dono, J., Martin, K., Bowden, J., & Miller, C. (2022). A population-level analysis of changes in Australian smokers' preferences for smoking cessation support over two decades - from 1998 to 2017. *LANCET REGIONAL HEALTH-WESTERN PACIFIC*, *19*. https://doi.org/doi:10.1016/j.lanwpc.2021.100342 | Only relative measures (e.g. Odds Ratios) |
| El Asmar, M. L., Laverty, A. A., Vardavas, C. I., & Filippidis, F. T. (2022). How do Europeans quit using tobacco, e-cigarettes and heated tobacco products? A cross-sectional analysis in 28 European countries. *BMJ OPEN*, *12*(4). https://doi.org/doi:10.1136/bmjopen-2021-059068 | No data on the use of mobile technology for smoking cessation |
| Ernsting, C., Dombrowski, S. U., Oedekoven, M., JL, O. S., Kanzler, M., Kuhlmey, A., & Gellert, P. (2017). Using Smartphones and Health Apps to Change and Manage Health Behaviors: A Population-Based Survey. *J Med Internet Res*, *19*(4), e101. https://doi.org/doi:10.2196/jmir.6838 | Wrong population (e.g. pre-selected by app use) |
| Etter, J. F. (2019). Are long-term vapers interested in vaping cessation support? *Addiction*, *114*(8), 1473-1477. https://doi.org/doi:10.1111/add.14595 | No data on the use of mobile technology for smoking cessation |
| Foxon, F., & Niaura, R. (2025). Use of nicotine products, prescription drug products, and other methods to stop smoking by US adults in the 2022 National Health Interview Survey. *INTERNAL AND EMERGENCY MEDICINE*, *20*(3), 691-700. https://doi.org/doi:10.1007/s11739-024-03847-6 | No data on the use of mobile technology for smoking cessation |
| Fujisawa, D., Umezawa, S., Basaki-Tange, A., Fujimori, M., & Miyashita, M. (2014). Smoking status, service use and associated factors among Japanese cancer survivors--a web-based survey. *Support Care Cancer*, *22*(12), 3125-3134. https://doi.org/doi:10.1007/s00520-014-2284-2 | No data on the use of mobile technology for smoking cessation |
| Gallaway, M. S., Glover-Kudon, R., Momin, B., Puckett, M., Lunsford, N. B., Ragan, K. R., Rohan, E. A., & Babb, S. (2019). Smoking cessation attitudes and practices among cancer survivors - United States, 2015. *JOURNAL OF CANCER SURVIVORSHIP*, *13*(1), 66-74. https://doi.org/doi:10.1007/s11764-018-0728-2 | No data on the use of mobile technology for smoking cessation |
| Gallaway, M. S., Huang, B., Chen, Q., Tucker, T. C., McDowell, J. K., Durbin, E., Stewart, S. L., & Tai, E. (2019). Smoking and Smoking Cessation Among Persons with Tobacco- and Non-tobacco-Associated Cancers. *JOURNAL OF COMMUNITY HEALTH*, *44*(3), 552-560. https://doi.org/doi:10.1007/s10900-019-00622-z | No data on the use of mobile technology for smoking cessation |
| Gambaryan, M., Kalinina, A., Popovich, M., Starovoytov, M., & Drapkina, O. (2019). Smoking cessation services in Russia: Do we meet the demand? results from Russian tobacco control policy evaluation survey. *European Respiratory Journal*, *54*. https://doi.org/doi:10.1183/13993003.congress-2019.OA5138 | Review, editorials, PhD theses, conference abstracts |
| Gambaryan, M., Kalinina, A. M., Popovich, M. V., Starovoytov, M. L., Drapkina, O. M., & Boytsov, S. A. (2019). Electronic cigarettes in Russia: Time for an action. Results from Russian Tobacco Control policy evaluation survey. *European Journal of Preventive Cardiology*, *26*, S113. https://doi.org/doi:10.1177/2047487319860053 | Review, editorials, PhD theses, conference abstracts |
| Gambaryan, M. G., Kalinina, A. M., Popovich, M. V., Starovoytov, M. L., Drapkina, O. M., Boytsov, S. A., & Salagay, O. O. (2019). The whole truth of electronic cigarettes: The russian reality. Part II. Use of electronic cigarettes in russia: Associations with demographic factors, advertisement and promotion, tobacco smoking and quit smoking attempts. Results from adult population representative survey EPOCHA-RF. *Profilakticheskaya Meditsina*, *22*(6), 14-27. https://doi.org/doi:10.17116/profmed20192206214 | No data on the use of mobile technology for smoking cessation |
| Gambaryan, M. H., Kalinina, A. M., Popovich, M. V., Starovoytov, M. L., Drapkina, O. M., & Boytsov, S. A. (2019). Demands for and implementation of smoking cessation support in health care: Results from Russian Tobacco Control Policy Evaluation Survey. *European Journal of Preventive Cardiology*, *26*, S114-S115. https://doi.org/doi:10.1177/2047487319860053 | Review, editorials, PhD theses, conference abstracts |
| Garey, L., Scott-Sheldon, L. A. J., Olofsson, H., Nelson, K. M., & Japuntich, S. J. (2021). Electronic cigarette cessation among adolescents and young adults. *Substance Use & Misuse*, *56*(12), 1900-1903. https://doi.org/doi:10.1080/10826084.2021.1958850 | No data on the use of mobile technology for smoking cessation |
| George, S., Becher, D., Zapor, M., & Hartzell, J. (2011). Smoking habits and cessation efforts among deployed soldiers. *Journal of General Internal Medicine*, *26*, S232. https://doi.org/doi:10.1007/s11606-011-1730-9 | Review, editorials, PhD theses, conference abstracts |
| Gifford, H., Wilson, D., Boulton, A., Walker, L., & Shepherd-Sinclair, W. (2013). Maori nurses and smoking: what do we know? *N Z Med J*, *126*(1384), 53-63. https://doi.org/doi: | No data on the use of mobile technology for smoking cessation |
| Gobarani, R. K., Zwar, N. A., Russell, G., Abramson, M. J., Bonevski, B., Holland, A. E., Paul, E., Cox, N. S., Wilson, S., & George, J. (2021). Smoking cessation intervention in Australian general practice: a secondary analysis of a duster randomised controlled trial. *BRITISH JOURNAL OF GENERAL PRACTICE*, *71*(707), E458-E464. https://doi.org/doi:10.3399/BJGP.2020.0906 | No data on the use of mobile technology for smoking cessation |
| Gore, E., Johnson, G., Nguyen, M. T. T., France, A., Perez-Colon, A. L., Bittner, K., Marino, D., & Kothari, S. (2022). PROMOTION OF SMOKING CESSATION FOR PATIENTS WITH CHRONIC PANCREATITIS: A QUALITY IMPROVEMENT INITIATIVE. *Gastroenterology*, *162*(7), S-862. https://doi.org/doi:10.1016/S0016-5085(22)62039-0 | Review, editorials, PhD theses, conference abstracts |
| Gorini, G., Carreras, G., Minardi, V., Masocco, M., Ferrante, G., Coppo, A., Gallus, S., Faggiano, F., Galeone, D., Spizzichino, L., Pacifici, R., & Vasselli, S. (2019). [Socioeconomic and regional inequalities in smoking cessation in Italy, 2014-2017]. *Epidemiol Prev*, *43*(4), 275-285. https://doi.org/doi:10.19191/ep19.4.P275.078 | No data on the use of mobile technology for smoking cessation |
| Graham, A. L., Milner, P., Saul, J. E., & Pfaff, L. (2008). Online advertising as a public health and recruitment tool: comparison of different media campaigns to increase demand for smoking cessation interventions. *J Med Internet Res*, *10*(5), e50. https://doi.org/doi:10.2196/jmir.1001 | No data on the use of mobile technology for smoking cessation |
| Guignard, R., Gallopel-Morvan, K., Mons, U., Hummel, K., & Nguyen-Thanh, V. (2018). Impact of a negative emotional antitobacco mass media campaign on French smokers: a longitudinal study. *Tob Control*, *27*(6), 670-676. https://doi.org/doi:10.1136/tobaccocontrol-2017-053936 | No data on the use of mobile technology for smoking cessation |
| Gutiérrez-Torres, D. S., Reyes-Guzman, C., Mayer, M., Prutzman, Y. M., & Freedman, N. D. (2025). Quit Attempts and Use of Cessation Aids Among U.S. Adults Who Smoke Nondaily. *AMERICAN JOURNAL OF PREVENTIVE MEDICINE*, *68*(3), 622-626. https://doi.org/doi:10.1016/j.amepre.2024.11.004 | No data on the use of mobile technology for smoking cessation |
| Henley, S. J., Asman, K., Momin, B., Gallaway, M. S., Culp, M. B., Ragan, K. R., Richards, T. B., & Babb, S. (2019). Smoking cessation behaviors among older US adults. *PREVENTIVE MEDICINE REPORTS*, *16*. https://doi.org/doi:10.1016/j.pmedr.2019.100978 | No data on the use of mobile technology for smoking cessation |
| Igarashi, A., Negishi, S., Goto, R., & Suwa, K. (2014). Web-based survey on smoking cessation behaviors of current and former smokers in Japan. *Curr Med Res Opin*, *30*(10), 1911-1921. https://doi.org/doi:10.1185/03007995.2014.938149 | No data on the use of mobile technology for smoking cessation |
| Jackson, S. E., Brown, J., & Beard, E. (2024). Associations of Prevalence of E-cigarette Use With Quit Attempts, Quit Success, Use of Smoking Cessation Medication, and the Overall Quit Rate Among Smokers in England: A Time-Series Analysis of Population Trends 2007-2022. *NICOTINE & TOBACCO RESEARCH*, *26*(7), 826-834. https://doi.org/doi:10.1093/ntr/ntae007 | No data on the use of mobile technology for smoking cessation |
| Jackson, S. E., Farrow, E., Brown, J., & Shahab, L. (2020). Is dual use of nicotine products and cigarettes associated with smoking reduction and cessation behaviours? A prospective study in England. *BMJ OPEN*, *10*(3). https://doi.org/doi:10.1136/bmjopen-2019-036055 | No data on the use of mobile technology for smoking cessation |
| Jackson, S. E., Garnett, C., Shahab, L., Oldham, M., & Brown, J. (2021). Association of the COVID-19 lockdown with smoking, drinking and attempts to quit in England: an analysis of 2019-20 data. *Addiction*, *116*(5), 1233-1244. https://doi.org/doi:10.1111/add.15295 | No data on the use of mobile technology for smoking cessation |
| Jackson, S. E., Kock, L., Kotz, D., & Brown, J. (2022). Real-world effectiveness of smoking cessation aids: A population survey in England with 12-month follow-up, 2015-2020. *ADDICTIVE BEHAVIORS*, *135*. https://doi.org/doi:10.1016/j.addbeh.2022.107442 | No data on the use of mobile technology for smoking cessation |
| Jeong, B. Y., Lim, M. K., Yun, E. H., & Oh, J. K. (2019). User characteristics of national smoking cessation services in Korea: who chooses each type of tobacco cessation program? *BMC Health Serv Res*, *19*(1), 14. https://doi.org/doi:10.1186/s12913-018-3817-z | Wrong population (e.g. pre-selected by app use) |
| Jeong, W., Kim, Y. K., Joo, J. H., Jang, S. I., & Park, E. C. (2020). The Association of Smoking Exposure at Home with Attempts to Quit Smoking and Cessation Success: A Survey of South Korean Adolescents Who Smoke. *Int J Environ Res Public Health*, *17*(11). https://doi.org/doi:10.3390/ijerph17114129 | No data on the use of mobile technology for smoking cessation |
| Kalkhoran, S., Kruse, G. R., Chang, Y. C., & Rigotti, N. A. (2018). Smoking-Cessation Efforts by US Adult Smokers with Medical Comorbidities. *AMERICAN JOURNAL OF MEDICINE*, *131*(3). https://doi.org/doi:10.1016/j.amjmed.2017.09.025 | No data on the use of mobile technology for smoking cessation |
| Kim, D. B., Park, Y. S., Yun, I., Park, E.-C., & Jang, S.-I. (2023). Association between anti-smoking campaign types and smoking cessation attempts. *SSM - Population Health*, *24*. https://doi.org/doi:10.1016/j.ssmph.2023.101505 | No data on the use of mobile technology for smoking cessation |
| Kim, D. J., Choo, E. K., & Ranney, M. L. (2013). Impact of gender on patient preferences for technology-based behavioral interventions. *Academic Emergency Medicine*, *20*(5), S180. https://doi.org/doi:10.1111/acem.12115 | No data on the use of mobile technology for smoking cessation |
| Klemperer, E. M., & Villanti, A. C. (2021). Why and how do dual users quit vaping? Survey findings from adults who use electronic and combustible cigarettes. *Tob Induc Dis*, *19*, 12. https://doi.org/doi:10.18332/tid/132547 | Sample size (<100 smokers) |
| Kulak, J. A., & LaValley, S. (2018). Cigarette use and smoking beliefs among older Americans: findings from a nationally representative survey. *J Addict Dis*, *37*(1), 46-54. https://doi.org/doi:10.1080/10550887.2018.1521255 | No data on the use of mobile technology for smoking cessation |
| Kumar, P., Gareen, I. F., Lathan, C., Sicks, J. D., Perez, G. K., Hyland, K. A., & Park, E. R. (2016). Racial Differences in Tobacco Cessation and Treatment Usage After Lung Screening: An Examination of the National Lung Screening Trial. *ONCOLOGIST*, *21*(1), 40-49. https://doi.org/doi:10.1634/theoncologist.2015-0325 | No data on the use of mobile technology for smoking cessation |
| Lawrance, K. G. (2001). Adolescent smokers' preferred smoking cessation methods. *Can J Public Health*, *92*(6), 423-426. https://doi.org/doi:10.1007/bf03404532 | No data on the use of mobile technology for smoking cessation |
| Leatherdale, S. T., & McDonald, P. W. (2005). What smoking cessation approaches will young smokers use? *ADDICTIVE BEHAVIORS*, *30*(8), 1614-1618. https://doi.org/doi:10.1016/j.addbeh.2005.02.004 | No data on the use of mobile technology for smoking cessation |
| Leatherdale, S. T., & Shields, M. (2009). Smoking cessation: intentions, attempts and techniques. *HEALTH REPORTS*, *20*(3). https://doi.org/doi: | No data on the use of mobile technology for smoking cessation |
| Lee, Y. S., Kim, H.-S., Kim, H.-D., Yoo, K.-B., Jang, S.-I., & Park, E.-C. (2016). Is a price increase policy enough for adolescent smokers?: Factors affecting the effectiveness of increasing cigarette prices among Korean adolescent smokers. *NICOTINE & TOBACCO RESEARCH*, *18*(10), 2013-2019. https://doi.org/doi:10.1093/ntr/ntw122 | No data on the use of mobile technology for smoking cessation |
| Liu, B., Zhan, S., Wilson, K. M., Mazumdar, M., & Li, L. H. (2021). The Influence of Increasing Levels of Provider-Patient Discussion on Quit Behavior: An Instrumental Variable Analysis of a National Survey. *INTERNATIONAL JOURNAL OF ENVIRONMENTAL RESEARCH AND PUBLIC HEALTH*, *18*(9). https://doi.org/doi:10.3390/ijerph18094593 | No data on the use of mobile technology for smoking cessation |
| Lopez-Olivo, M. A., Michaud, K., Schumacher, R., Minnix, J., Cinciripini, P., & Suarez-Almazor, M. E. (2023). Smoking cessation patterns, usefulness of quitting methods, and tobacco cessation motivators and barriers to quit in patients with rheumatoid arthritis. *CLINICAL RHEUMATOLOGY*, *42*(8), 2053-2068. https://doi.org/doi:10.1007/s10067-023-06593-w | No data on the use of mobile technology for smoking cessation |
| Lund, M., & Kvaavik, E. (2021). Methods Used in Smoking Cessation and Reduction Attempts: Findings from Help-Seeking Smokers. *J Smok Cessat*, *2021*, 6670628. https://doi.org/doi:10.1155/2021/6670628 | Wrong population (e.g. pre-selected by app use) |
| Mai, Y., & Soulakova, J. N. (2018). Retrospective reports of former smokers: Receiving doctor's advice to quit smoking and using behavioral interventions for smoking cessation in the United States. *Prev Med Rep*, *11*, 290-296. https://doi.org/doi:10.1016/j.pmedr.2018.07.012 | No data on the use of mobile technology for smoking cessation |
| Maxwell, A. E., Garcia, G. M., & Berman, B. A. (2007). Understanding tobacco use among Filipino American men. *Nicotine and Tobacco Research*, *9*(7), 769-776. https://doi.org/doi:10.1080/14622200701397890 | No data on the use of mobile technology for smoking cessation |
| Michell, C., & Oakeshott, P. (2014). Text messaging to promote health. *Br J Gen Pract*, *64*(619), 72-73. https://doi.org/doi:10.3399/bjgp14X677086 | Review, editorials, PhD theses, conference abstracts |
| Muñoz, R. F., Chen, K., Bunge, E. L., Bravin, J. I., Shaughnessy, E. A., & Pérez-Stable, E. J. (2014). Reaching Spanish-speaking smokers online: a 10-year worldwide research program. *Rev Panam Salud Publica*, *35*(5), 407-414. https://doi.org/doi: | Wrong context (no high-income country) |
| Neumann, M. F., Murphy, N. J., & Seetharamu, N. (2022). Smoking cessation after cancer diagnosis: Relationships matter. *Journal of Clinical Oncology*, *40*(16). https://doi.org/doi:10.1200/JCO.2022.40.16_suppl.e24039 | Review, editorials, PhD theses, conference abstracts |
| Onyeaka, H. K., Chido-Amajuoyi, O. G., Daskalakis, E., Deary, E. C., Boardman, A. C., Basiru, T., Muoghalu, C., Uwandu, Q., Baiden, P., Nkemjika, S., Aneni, K., & Amonoo, H. L. (2024). Associations between Health-Related Use of Social Media and Positive Lifestyle Behaviors: Findings from a Representative Sample of US Adult Smokers. *Subst Use Misuse*, *59*(4), 527-535. https://doi.org/doi:10.1080/10826084.2023.2287199 | No data on the use of mobile technology for smoking cessation |
| Onyeaka, H. K., Zambrano, J., Longley, R. M., Celano, C. M., Naslund, J. A., & Amonoo, H. L. (2021). Use of digital health tools for health promotion in cancer survivors. *Psycho-Oncology*, *30*(8), 1302-1310. https://doi.org/doi:10.1002/pon.5677 | No data on the use of mobile technology for smoking cessation |
| Otachi, J. K., Seng, S., & Okoli, C. T. C. (2020). Factors associated with tobacco cessation attempts among inpatients in a psychiatric hospital. *JOURNAL OF SMOKING CESSATION*, *15*(2), 84-93. https://doi.org/doi:10.1017/jsc.2020.3 | No data on the use of mobile technology for smoking cessation |
| Pettigrew, S., Jun, M., Roberts, I., Bullen, C., Nallaiah, K., & Rodgers, A. (2020). Preferences for tobacco cessation information and support during Covid-19. *Journal of Addiction Medicine*, *14*(6), E362-E365. https://doi.org/doi:10.1097/ADM.0000000000000743 | No data on the use of mobile technology for smoking cessation |
| Prochaska, J. J., Reyes, R. S., Schroeder, S. A., Daniels, A. S., Doederlein, A., & Bergeson, B. (2011). An online survey of tobacco use, intentions to quit, and cessation strategies among people living with bipolar disorder. *Bipolar Disord*, *13*(5), 466-473. https://doi.org/doi:10.1111/j.1399-5618.2011.00944.x | No data on the use of mobile technology for smoking cessation |
| Quinn, M. H., Olonoff, M., Bauer, A. M., Fox, E., Jao, N., Lubitz, S. F., Leone, F., Gollan, J. K., Schnoll, R., & Hitsman, B. (2022). History and Correlates of Smoking Cessation Behaviors Among Individuals With Current or Past Major Depressive Disorder Enrolled in a Smoking Cessation Trial. *NICOTINE & TOBACCO RESEARCH*, *24*(1), 37-43. https://doi.org/doi:10.1093/ntr/ntab147 | No data on the use of mobile technology for smoking cessation |
| Rodu, B., & Plurphanswat, N. (2017). Quit Methods Used by American Smokers, 2013-2014. *INTERNATIONAL JOURNAL OF ENVIRONMENTAL RESEARCH AND PUBLIC HEALTH*, *14*(11). https://doi.org/doi:10.3390/ijerph14111403 | No data on the use of mobile technology for smoking cessation |
| Ruggiero, K. J., Gros, D. F., McCauley, J., de Arellano, M. A., & Danielson, C. K. (2011). Rural adults' use of health-related information online: data from a 2006 National Online Health Survey. *Telemed J E Health*, *17*(5), 329-334. https://doi.org/doi:10.1089/tmj.2010.0195 | Wrong population (e.g. pre-selected by app use) |
| Rutqvist, L. E. (2012). Population-based survey of cessation aids used by Swedish smokers. *HARM REDUCTION JOURNAL*, *9*. https://doi.org/doi:10.1186/1477-7517-9-38 | No data on the use of mobile technology for smoking cessation |
| Salisbury-Afshar, E., & Fitzgerald, R. M. (2020). Smoking Cessation with Text Messaging and App-Based Interventions. *American Family Physician*, *102*(3), 148-149. https://doi.org/doi: | Review, editorials, PhD theses, conference abstracts |
| Sazegar, P., Martinez, A. N., & Varallo, M. D. (2023). Smoking cessation in adolescents. *American Family Physician*, *107*(5), 450E-451E. https://doi.org/doi: | Review, editorials, PhD theses, conference abstracts |
| Shiffman, S., Brockwell, S. E., Pillitteri, J. L., & Gitchell, J. G. (2008). Use of smoking-cessation treatments in the United States. *AMERICAN JOURNAL OF PREVENTIVE MEDICINE*, *34*(2), 102-111. https://doi.org/doi:10.1016/j.amepre.2007.09.033 | No data on the use of mobile technology for smoking cessation |
| Shiffman, S., Brockwell, S. E., Pillitteri, J. L., & Gitchell, J. G. (2008). Individual differences in adoption of treatment for smoking cessation: Demographic and smoking history characteristics. *DRUG AND ALCOHOL DEPENDENCE*, *93*(1), 121-131. https://doi.org/doi:10.1016/j.drugalcdep.2007.09.005 | No data on the use of mobile technology for smoking cessation |
| Smit, E. S., Hoving, C., Cox, V. C., & de Vries, H. (2012). Influence of recruitment strategy on the reach and effect of a web-based multiple tailored smoking cessation intervention among Dutch adult smokers. Health Educ Res, 27(2), 191-199. https://doi.org/doi:10.1093/her/cyr099 | No data on the use of mobile technology for smoking cessation |
| Smit, E. S., Hoving, C., Schelleman-Offermans, K., West, R., & de Vries, H. (2014). Predictors of successful and unsuccessful quit attempts among smokers motivated to quit. Addict Behav, 39(9), 1318-1324. https://doi.org/doi:10.1016/j.addbeh.2014.04.017 | No data on the use of mobile technology for smoking cessation |
| Smith, P., Daniel, R., Murray, R. L., Moore, G., Nelson, A., & Brain, K. (2021). Psychosocial determinants of quit motivation in older smokers from deprived backgrounds: A cross-sectional survey. BMJ OPEN, 11(5). https://doi.org/doi:10.1136/bmjopen-2020-044815 | No data on the use of mobile technology for smoking cessation |
| Solberg, L. I., Boyle, R. G., McCarty, M., Asche, S. E., & Thoele, M. J. (2007). Young adult smokers: Are they different? AMERICAN JOURNAL OF MANAGED CARE, 13(11), 626-632. https://doi.org/doi: | No data on the use of mobile technology for smoking cessation |
| Sousa, R., & Moreira, C. (2015). Smoking cessation in primary care. The experience of a general practice unit. *Journal of Hypertension*, *33*, e147. https://doi.org/doi:10.1097/01.hjh.0000467770.35090.8b | Review, editorials, PhD theses, conference abstracts |
| Squiers, L., Brown, D., Parvanta, S., Dolina, S., Kelly, B., Dever, J., Southwell, B. G., Sanders, A., & Augustson, E. (2016). The SmokefreeTXT (SFTXT) Study: Web and Mobile Data Collection to Evaluate Smoking Cessation for Young Adults. *JMIR Res Protoc*, *5*(2), e134. https://doi.org/doi:10.2196/resprot.5653 | No data on the use of mobile technology for smoking cessation |
| Stoddard, J., Delucchi, K., Muñoz, R., Collins, N., Stable, E. P., Augustson, E., & Lenert, L. (2005). Smoking cessation research via the internet: a feasibility study. *J Health Commun*, *10*(1), 27-41. https://doi.org/doi:10.1080/10810730590904562 | No data on the use of mobile technology for smoking cessation |
| Stoddard, J. L., & Augustson, E. M. (2006). Smokers who use Internet and smokers who don't: Data from the Health Information and National Trends Survey (HINTS). *NICOTINE & TOBACCO RESEARCH*, *8*, S77-S85. https://doi.org/doi:10.1080/14622200601039147 | No data on the use of mobile technology for smoking cessation |
| Sweeney, M. M., Holtyn, A. F., Stitzer, M. L., & Gastfriend, D. R. (2022). Practical Technology for Expanding and Improving Substance Use Disorder Treatment: Telehealth, Remote Monitoring, and Digital Health Interventions. *Psychiatric Clinics of North America*, *45*(3), 515-528. https://doi.org/doi:10.1016/j.psc.2022.05.006 | Review, editorials, PhD theses, conference abstracts |
| Tabuchi, T. (2021). ES24.03 Current Status of Smoking Cessation with Heated Tobacco Products Use in Japan. *Journal of Thoracic Oncology*, *16*(3), S91. https://doi.org/doi:10.1016/j.jtho.2021.01.053 | Review, editorials, PhD theses, conference abstracts |
| Takahashi, Y., Satomura, K., Miyagishima, K., Nakahara, T., Higashiyama, A., Iwai, K., & Nakamura, K. (1999). A new smoking cessation programme using the Internet. *Tobacco control*, *8*(1), 109-110. https://doi.org/doi:10.1136/tc.8.1.109-b | Review, editorials, PhD theses, conference abstracts |
| Tanaka, S., Ihira, H., Tajima, T., Kanehara, R., Inoue, Y., Doi, T., Kobayashi, N., Kato, Y., Nishita, Y., Konishi, M., Nakao, Y. M., Kabe, I., Morioka, C., Dohi, S., Khairan, P., Arafa, A., Narita, Z. C., Morisaki, N., Lu, Y.,…Inoue, M. (2024). *Birth Cohort-Specific Smoking Patterns in Japan (1906-2004): Insights from the NC-CCAPH Consortium*. https://doi.org/doi:10.2139/ssrn.5070291 | No data on the use of mobile technology for smoking cessation |
| Taylor, D., Bradley, E., Meng, S., Thapa, J., & Goldenberg, E. (2016). Tobacco Cessation for Patients with Coronary Artery Disease. *Delaware medical journal*, *88*(3), 84-88. https://doi.org/doi: | Review, editorials, PhD theses, conference abstracts |
| Tong, V. T., England, L. J., Dietz, P. M., & Asare, L. A. (2008). Smoking Patterns and Use of Cessation Interventions During Pregnancy. *AMERICAN JOURNAL OF PREVENTIVE MEDICINE*, *35*(4), 327-333. https://doi.org/doi:10.1016/j.amepre.2008.06.033 | No data on the use of mobile technology for smoking cessation |
| Tregobov, N., Poureslami, I., Ip, J., & FitzGerald, M. (2019). Supplementing smoking-cessation practices amongst cantonese and mandarin speaking smokers through the investigation of perceptions, attitudes and beliefs. *Canadian Journal of Respiratory, Critical Care, and Sleep Medicine*, *3*, 48-49. https://doi.org/doi:10.1080/24745332.2019.1623590 | Review, editorials, PhD theses, conference abstracts |
| Tsiouda, T., Zarogoulidis, P., Petridis, D., Pezirkianidis, N., Kioumis, I., Yarmus, L., Huang, H., Li, Q., Hohenforst-Schmidt, W., Porpodis, K., Spyratos, D., Tsakiridis, K., Pitsiou, G., Kontakiotis, T., Argyropoulou, P., Kyriazis, G., & Zarogoulidis, K. (2014). A multifactoral analysis of 1452 patients for smoking sensation. An outpatient lab experience. *Journal of Cancer*, *5*(6), 433-445. https://doi.org/doi:10.7150/jca.9360 | No data on the use of mobile technology for smoking cessation |
| Ustun, G., Söylemez, S. L., Ucar, N., Sancar, M., & Okuyan, B. (2020). E-health literacy and mobile health application utilization among pharmacy students. *International Journal of Clinical Pharmacy*, *42*(1), 291. https://doi.org/doi:10.1007/s11096-019-00945-w | Review, editorials, PhD theses, conference abstracts |
| Uwandu, Q., Onyeaka, H. K., Akpoviroro, O., Chido-Amajuoyi, O., Nwadukwe, I., Onyeaka, C. N., & Omede, F. F. (2023). Social Media for Smoking Cessation among Adult Smokers in the United States. *Journal of Addiction Medicine*, *17*(5), e343-e344. https://doi.org/doi: | Review, editorials, PhD theses, conference abstracts |
| Walter, K. (2021). Ways to Quit Smoking. *JAMA - Journal of the American Medical Association*, *326*(1), 96. https://doi.org/doi:10.1001/jama.2021.7239 | Review, editorials, PhD theses, conference abstracts |
| Watkins, S. L., Thrul, J., Max, W., & Ling, P. M. (2020). Cold Turkey and Hot Vapes? A National Study of Young Adult Cigarette Cessation Strategies. *NICOTINE & TOBACCO RESEARCH*, *22*(5), 638-646. https://doi.org/doi:10.1093/ntr/nty270 | No data on the use of mobile technology for smoking cessation |
| Weld-Blundell, I., Grech, L., Borland, R., White, S. L., das Nair, R., & Marck, C. H. (2022). Smoking habits, awareness and support needs for cessation among people with multiple sclerosis in Australia: findings from an online survey. *BMJ OPEN*, *12*(7), e059637. https://doi.org/doi:10.1136/bmjopen-2021-059637 | Sample size (<100 smokers) |
| West, R., Gilsenan, A., Coste, F., Zhou, X., Brouard, R., Nonnemaker, J., Curry, S. J., & Sullivan, S. D. (2006). The ATTEMPT cohort: A multi-national longitudinal study of predictors, patterns and consequences of smoking cessation; introduction and evaluation of internet recruitment and data collection methods. *Addiction*, *101*(9), 1352-1361. https://doi.org/doi:10.1111/j.1360-0443.2006.01534.x | No data on the use of mobile technology for smoking cessation |
| Wiium, N., Overland, S., & Aarø, L. E. (2011). Smoking cessation among Norwegian adolescents and young adults: preferred cessation methods. *Scand J Psychol*, *52*(2), 154-160. https://doi.org/doi:10.1111/j.1467-9450.2010.00851.x | No data on the use of mobile technology for smoking cessation |
| Wilson, J. F. (2007). In the clinic. Smoking cessation. *Ann Intern Med*, *146*(3), Itc2-1-ict2-16. https://doi.org/doi:10.7326/0003-4819-146-3-200702060-01002 | Review, editorials, PhD theses, conference abstracts |
| Yeomans, K., Payne, K. A., Marton, J. P., Merikle, E. P., Proskorovsky, I., Zou, K. H., Li, Q., & Willke, R. J. (2011). Smoking, smoking cessation and smoking relapse patterns: A web-based survey of current and former smokers in the US. *International Journal of Clinical Practice*, *65*(10), 1043-1054. https://doi.org/doi:10.1111/j.1742-1241.2011.02758.x | No data on the use of mobile technology for smoking cessation |
| Zhou, X., Nonnemaker, J., Sherrill, B., Gilsenan, A. W., Coste, F., & West, R. (2009). Attempts to quit smoking and relapse: Factors associated with success or failure from the ATTEMPTS cohort study. *ADDICTIVE BEHAVIORS*, *34*(4), 365-373. https://doi.org/doi:10.1016/j.addbeh.2008.11.013 | No data on the use of mobile technology for smoking cessation |
| Zhu, S. H., Melcer, T., Sun, J. C., Rosbrook, B., & Pierce, J. P. (2000). Smoking cessation with and without assistance - A population-based analysis. *AMERICAN JOURNAL OF PREVENTIVE MEDICINE*, *18*(4), 305-311. https://doi.org/doi:10.1016/S0749-3797(00)00124-0 | No data on the use of mobile technology for smoking cessation |

**sTable 4: Studies definition of smokers**

| **Authors & Year** | **Country** | **Definition smokers / smoking** |
| --- | --- | --- |
| Smoking cessation |  |  |
| Borrelli et al. 2015 | US/UK | Smoked at least 100 cigarettes in their lifetime and three cigarettes per day for the past year |
| Bottorff et al. 2016 | Canada | Smoking daily or occasionally |
| Caraballo et al. 2017 | USA | Smoked at least 100 cigarettes in their lifetime and smoke daily or some days |
| Chevalking et al. 2018 | Netherlands | Smokers: Smoking regularly;  Ex-smokers: Smoked regularly but have quit;  Smokers who are currently attempting to quit were registered as ex-smokers |
| Curry et al. 2007  Companion report:  Coups et al. 2009 | USA | Smoked at least 100 cigarettes in their lifetime, smoking every day or some days |
| Graham et al. 2019 | USA | Smoked at least 100 cigarettes in life, smoking every day or some days |
| Gravely et al. 2021 | Australia, Canada, England, USA | Smoked at least 100 cigarettes in their lifetime, smoking daily, vaping more than weekly or daily smoking in the past |
| Hummel et al. 2018 | England, Germany, Greece, Hungary, Netherlands, Poland, Romania, Spain | Smoked at least 100 cigarettes in their lifetime, currently smoking cigarettes at least monthly |
| Jackson et al. 2019 | UK | Smoking any tobacco product daily or occasionally at the time of the survey or during the preceding 12 months |
| Jackson et al. 2022 | UK | Smoking any tobacco product daily or occasionally at the time of the survey or during the preceding 12 months |
| Jackson et al. 2025 | UK | Smoking any tobacco product daily or occasionally at the time of the survey or during the preceding 12 months |
| Jayakumar et al. 2020 | Canada | Smoking daily or occasionally, or former smoking |
| Kostagiolas et al. 2023 | Greece | N/R |
| Li et al. 2025 | Australia | Smoking daily |
| Lund & Lund 2022 | Norway | Smoking daily or formerly daily smoking |
| Lund & Lund 2023 | Norway | Smoking daily or occasionally |
| Moon et al. 2020 | USA | N/R |
| Oliver et al. 2018 | USA | Smoking daily (≥5 cigarettes per day) |
| Papadakis et al. 2020 | England, Germany, Greece, Hungary, Poland, Romania, Spain | N/R |
| Patterson et al. 2021 | USA | Smoking daily or some days/week (combustible cigarettes, smokeless tobacco, e-cigarettes or several |
| Perski et al. 2022  Companion reports:  Beard et al. 2016,  Perski et al. 2019 | UK | N/R |
| Ramo et al. 2015 | USA | Smoked at least once in the past month |
| Thrul et al. 2021  Companion reports:  Mojtabai et al. 2020,  Soulakova & Crockett 2016 | US | N/R |
| Tofighi et al, 2019 | US | N/R |
| Twyman et al. 2018 | Australia | Smoked at least 100 cigarettes or a similar amount of tobacco in their life and smoking daily, once a week or less often than once a week |
| Vaping Cessation |  |  |
| Dai et al. 2023 | USA | Using e-cigarettes ≥1 day(s) in the past 30 days |
| Jones et al. 2023 | USA | Vaped >= 1 day in the last 30 Days (current vapers) or did not vape in the last 30 Days (former vapers) |

**sTable 5: Prevalence of the use of different mobile technology tools for smoking cessation among smokers and former smokers, stratified by sex/age**

| **Authors & Year** | **Country** | **Year of Data** | **Type of mobile technology for smoking cessation** | **Stratified prevalence by sex**  **(% (n))** | **Stratified prevalence by age** |
| --- | --- | --- | --- | --- | --- |
| Lifetime prevalences | | | | | |
| Jayakumar et al. 2020 | Canada; Smokers and former smokers aged 18 years and older | 2013-2018 | Apps | Female (n=1,765): 47 (2.7%)  Male (n=1,008): 22 (2.2%) | N/R |
| Period prevalences | | | | | |
| Patterson et al. 2021 | USA; Smokers aged 18 and older | 2018-2019 | Internet/web-based program  Videos | Female (n=280):  Internet/ web-based program: 4 (1.4%)  Videos: 3 (1.1%)  Male (n=379):  Internet/ web-based program: 7 (1.8%)  Videos: 2 (0.5%) | N/R |
| Thrul et al. 2021 | US; Smokers aged 18 and older | 2010-2011 | Internet/websites/ web-based programs | Female (n=13,785): 148 (1.1%) male (n= 13,538): 88 (0.6%) | 18-29 years (n=5,309): 64 (1.2%)  30-49 years (n=11,242): 96 (0.7%)  50-64 years (n=8,080): 63 (0.7%)  65+ (n=2,696): 13 (0.5%) |
| Point prevalences | | | | | |
| Beard et al. 2016 | UK | 2014-2015 | Website or apps | Female: 1.3% (n=819)  Male: 1.5%ᵃ (n=781) | N/R |
| Curry et al. 2007 | USA | 2005 | Internet | N/R | 18-24 years old (n=387): 3.6%¹  >25 years old (n=2,360): 2.9%¹ |
| Hummel et al. 2018 | England  Germany  Greece  Hungary  Netherlands  Poland  Romania  Spain | 2016 | Internet | England: Female: 7.3%¹  Male: 14.4%¹  Germany: Female: 2.9%¹  Male: 6.6%¹  Greece: Female: 0.8%¹  Male: 0¹  Hungary: Female: 0¹  Male: 5.3%¹  Netherlands: Female: 6.3%¹  Male: 5.3%¹  Poland: Female: 3.3%¹  Male: 7.7%¹  Romania: Female: 3.0%¹  Male: 3.3%¹  Spain: Female: 0¹  Male: 0¹ | England: 18-24 years: 20.7%¹  25-39 years: 13.5%¹  40-54 years: 7.3%¹  55+ years: 1.6%¹  Germany: 18-24 years: 0¹  25-39 years: 7.1%¹  40-54 years: 5.5%¹  55+ years: 3.0%¹  Greece: 18-24 years: 0¹  25-39 years: 0¹  40-54 years: 1.2%¹  55+ years: 0¹  Hungary: 18-24 years: 0¹  25-39 years: 0¹  40-54 years: 0¹  55+ years: 12.3%¹  Netherlands: 18-24 years: 3.0%¹  25-39 years: 9.2%¹  40-54 years: 6.6%¹  55+ years: 3.3%¹  Poland: 18-24 years: 12.1%¹  25-39 years: 8.3%¹  40-54 years: 2.8%¹  55+ years: 0¹  Romania: 18-24 years: 12.1%¹  25-39 years: 0.6%¹  40-54 years: 3.7%¹  55+ years: 0¹  Spain: 18-24 years: 0¹  25-39 years: 0¹  40-54 years: 0¹  55+ years: 0¹ |
| Jackson et al. 2020 | UK; Smokers and former smokers aged 16 and older who made at least one quit attempt in the past year | 2006- 2018 | Websites | Female (n=9,845): 96 (1.0%)ᵇ  Male (n=9,084): 108 (1.2%)ᶜ | 16-24 years (n=3,862): 52 (1.3%)ᵈ  25-34 years (n=4,403): 64 (1.5%)ᵉ  35-44 years (n=3,815): 41 (1.1%)ᶠ  45-54 year (n=2,994): 26 (0.9%)ᵍ  55-64 years (n=2,207): 16 (0.7%)ʰ  65+ years (n=1,648): 5 (0.3%)ⁱ |
| Lund & Lund 2023 | Norway; Smokers and former smokers aged | 2020 | Apps | Female (n=587): 88ʲ (15.0%)  Male (n=524): 31ᵏ (5.9%) | 18-34 years (n=250): 50ˡ (20.0%)  >35 years (n=861): 69ᵐ (8.0%) |

**sTable 6: Prevalence of the use and intention to use of different mobile technology tools for vaping cessation among vapers, stratified by prevalence type**

| **Authors & Year** | **Country** | **Year of Data** | **Type of mobile technology for smoking cessation** | **Prevalence of mobile technology use for smoking cessation (% (n))** | **Prevalence of intention to use mobile technologies in the future (% (n))** |
| --- | --- | --- | --- | --- | --- |
| Lifetime prevalences | | | | | |
| Jones et al. 2023 | USA | N/R | Text messaging program  Social media (TikTok, Instagram, Youtube, Snapchat, Twitter, Facebook, Pinterest)  Apps  Online/web-based programs | Text messaging program: 17.3%  TikTok: 12.4%ᵍ  Instagram: 11.9%ʰ  Youtube: 7.0%ⁱ  Snapchat: 6.5%ʲ  Twitter: 2.2%ᵏ  Facebook: 1.1%ˡ  Pinterest: 1.1%ᵐ  Apps: 12.4%  Online/web-based programs: 9.7%  (n=185) | Text message program: 30.8%  Social media (Facebook, Instagram, TikTok, other): 22.8%  Mobile apps: 34.6%  Online or web-based program (excluding social media): 9.7%  (n=185) |
| Period prevalence (past year) | | | | | |
| Dai et al. 2023 | USA | 2021 | Internet  Apps or text messaging program | Internet: 6.3%^p^  Apps or text messaging program: 5.5%^q^  (n=889) | N/R |
